# Dizygotic Atomic Platinum and Palladium on Carbon for High‐Performance Ethanol and Methanol Electro‐Oxidation

**DOI:** 10.1002/anie.202502348

**Published:** 2025-07-22

**Authors:** Zhiqi Zhang, Jiapeng Liu, Shangqian Zhu, Yuhao Wang, Jian Wang, Minhong Xu, Jie Zhao, Zheng Wang, Dewang Zeng, Jianrong Zeng, Yufei Song, Chih‐Wen Pao, Zhiwei Hu, Jongwoo Lim, Rui Xiao, Minhua Shao, Francesco Ciucci

**Affiliations:** ^1^ Key Laboratory of Energy Thermal Conversion and Control (Ministry of Education) School of Energy and Environment Southeast University Nanjing P.R. China; ^2^ School of Advanced Energy Sun‐Yat Sen University Shenzhen P.R. China; ^3^ Department of Mechanical and Aerospace Engineering The Hong Kong University of Science and Technology Hong Kong P.R. China; ^4^ Department of Chemical and Biological Engineering The Hong Kong University of Science and Technology Hong Kong P.R. China; ^5^ School of Chemistry and Chemical Engineering Southeast University Nanjing P.R. China; ^6^ School of Energy and Environment City University of Hong Kong Kowloon Hong Kong P.R. China; ^7^ Institute for Advanced Study Shenzhen University Shenzhen 518060 P.R. China; ^8^ Shanghai Synchrotron Radiation Facility Shanghai Advanced Research Institute Chinese Academy of Sciences Shanghai 201204 P.R. China; ^9^ Shanghai Institute of Applied Physics Chinese Academy of Sciences Shanghai 201800 P.R. China; ^10^ National Synchrotron Radiation Research Center 101 Hsin‐Ann Road Taiwan Beamline 44 Hsinchu 30076 Taiwan; ^11^ Max‐Planck‐Institute for Chemical Physics of Solids Nöthnitzer Str. 40 Dresden 01187 Germany; ^12^ Department of Chemistry College of Science Seoul National University Seoul 08826 South Korea; ^13^ Energy Institute The Hong Kong University of Science and Technology Hong Kong P.R. China; ^14^ Chair of Electrode Design for Electrochemical Energy Systems University of Bayreuth Weiherstraße 26 95448 Bayreuth Bavaria Germany; ^15^ Bavarian Center for Battery Technology (BayBatt) University of Bayreuth Universitätsstraße 30 95447 Bayreuth Bavaria Germany

**Keywords:** Alcohol electrooxidation, Dizygotic‐atom‐site catalysts, Ethanol electrooxidation, Methanol electrooxidation, Single atom catalysts

## Abstract

Dizygotic‐atom‐site catalysts (DASCs), consisting of multi‐atomic dispersed catalytic centers, perform well in several reactions but have poor electrocatalytic activity toward alcohol electro‐oxidation. In this study, DASCs of atomically dispersed platinum and palladium on nitrogen‐doped carbon nanocages (Pt_1_Pd_1_/NCNC) are successfully synthesized using an impregnation–adsorption method. The Pt_1_Pd_1_/NCNC catalyst has higher mass activity toward ethanol and methanol oxidation than commercial Pt/C and Pd/C. In contrast, Pt or Pd single‐atom catalysts on nitrogen‐doped carbon nanocages are virtually inert. Ethanol and methanol on Pt_1_Pd_1_/NCNC are electro‐oxidized to acetate ion and CO_2_ as the final product, respectively. Pt_1_Pd_1_/NCNC exhibits long‐term stability toward ethanol and methanol oxidation due to the absence of the CO intermediate. Ab initio simulations show that Pt_1_Pd_1_/NCNC optimizes the ethanol oxidation pathway thanks to a lower energy barrier and onset potential than individual single Pt or Pd atom catalysts on NCNC. This study opens a new path to developing advanced DASCs for alcohol oxidation.

## Graphical abstract

Single‐atom catalysts (SACs) have been explored to maximize Pt utilization, but they struggle to efficiently activate the C─H and O─H bonds in alcohols. Multi‐metallic dizygotic‐atom‐site catalysts (DASCs), which incorporate multiple metal atoms in dispersed active sites, offer a promising alternative by enhancing catalytic activity through metal synergy. Pt and Pd, with similar electronic properties, can work synergistically in DASCs to improve alcohol electro‐oxidation efficiency.

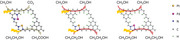

## Introduction

Pt is the most active electrocatalyst for the anodic alcohol oxidation reaction (AOR), including the ethanol oxidation reaction (EOR) and methanol oxidation reaction (MOR), but suffers from high cost and scarcity.^[^ ^1^, ^2^, ^3^ ^]^ Meanwhile, Pt bulk/nanoparticle catalysts are highly susceptible to poisoning by CO intermediates generated during AOR, leading to inferior stability. Thus, improving the electrocatalytic activity and durability while reducing Pt use is a long‐term goal of the AOR field.^[^ ^4^ ^]^ One of the most promising strategies to achieve this goal is to maximize the utilization of every Pt atom using single‐atom catalysts (SACs). Unfortunately, carbon‐supported Pt single atoms are ineffective in triggering the electrochemical dehydrogenation of methanol^[^ ^5^, ^6^ ^]^ as the AOR involves the cleavage of one O─H bond and multiple C─H bonds. Therefore, a single Pt atom center does not perform well in catalyzing this reaction.^[^ ^7^ ^]^

To make Pt SACs more active toward the AOR, the active center's chemical environment can be adjusted. For instance, it has been shown that Pt single atoms are excellent MOR electrocatalysts if coordinated onto RuO_2_ but are inert on carbon.^[^ ^8^ ^]^ However, due to Ru's scarcity and high cost, the practical applications of this electrocatalyst are limited. Hence, designing atomically active catalysts centered on low‐cost carbon as highly functional materials for AOR is both meaningful and challenging.

Dizygotic‐atom‐site catalysts (DASCs) with multi‐atomic dispersed catalytic centers have found applications in homogeneous and heterogeneous catalysis.^[^ ^9^ ^]^ Similar to SACs, DASCs feature unsaturated coordination bonds of the active centers, unique electronic structures, and utmost utilization of precious metal atoms. Multi‐metallic atoms in DASCs have been shown to provide hetero‐metal active centers and favorable interactions between active sites, leading to improved or even unexpectedly high intrinsic activity.^[^ ^10^ ^]^ For instance, the catalytic activity toward many electrocatalytic reactions except AOR is significantly enhanced for Ni_1_Fe_1_,^[^ ^11^, ^12^, ^13^, ^14^, ^15^ ^]^ Co_1_Zn_1_,^[^ ^16^, ^17^ ^]^ Fe_1_Co_1_,^[^ ^18^ ^]^ Mo_1_W_1_,^[^ ^10^ ^]^ Co_1_Ni_1_,^[^ ^19^ ^]^ Ni_1_Zn_1_,^[^ ^20^ ^]^ Pd_1_Cu_1_,^[^ ^21^ ^]^ and Pt_1_Ru_1_^[^ ^22^ ^]^ relative to individual SACs. In this regard, by leveraging dizygotic metal site synergy, DASCs are expected to be better candidates than SACs for activating O─H and C─H cleavage during AOR. However, DASCs have yet to be used in AOR.

Pd and Pt share similar properties due to their face‐centered cubic crystal structures, similar atomic sizes, and situation in the same group in the periodic table.^[^ ^23^ ^]^ In fact, Pd is the other element, besides Pt, that demonstrates effective electrochemical activity for AOR, exhibiting comparable performance in alkaline solutions.^[^ ^3^, ^24^ ^]^ Notably, Pd favors the direct electro‐oxidation of HCOOH to CO_2_, bypassing the production of CO as an intermediate.^[^ ^24^ ^]^ The absence of CO prevents catalyst poisoning, implying an enhancement, in principle, of AOR efficiency. Hence, these considerations suggest that a Pd single‐atom center combined with a Pt single‐atom center would be an ideal candidate for synergistically catalyzing AOR.

In this study, we constructed dizygotic Pt and Pd atoms on hierarchical nitrogen‐doped carbon nanocages (NCNC) (Pt_1_Pd_1_/NCNC) and Pt/Pd single atoms on NCNC (Pt_1_/NCNC, Pd_1_/NCNC) using a simple impregnation–adsorption method.^[^ ^25^ ^]^ The Pt_1_Pd_1_/NCNC showed high mass activity toward the EOR (2692.5 ± 50.4 mA mg^‒1^_metal_) and MOR (2578.6 ± 35.8 mA mg^‒1^_metal_), far better than Pt_1_/NCNC and Pd_1_/NCNC, two virtually inert compounds. Meanwhile, Pt_1_Pd_1_/NCNC displayed higher long‐term EOR and MOR stability compared to commercial Pt/C and Pd/C. This enhanced stability is attributed to the absence of CO intermediates during EOR and MOR on Pt_1_Pd_1_/NCNC. The corresponding EOR and MOR mechanisms on Pt_1_Pd_1_/NCNC were revealed by combining *operando* infrared reflection absorption spectroscopy (IRRAS) experiments with insights from density functional theory (DFT) simulations. In particular, Pt_1_Pd_1_/NCNC favors the electro‐oxidation of ethanol to acetate as the final product with a lower energy barrier and onset potential than Pt_1_/NCNC and Pd_1_/NCNC. This study provides an approach to exploring advanced DASCs toward AOR by multi‐active centers.

## Results and Discussion

### Structure Characterization of Pt_1_Pd_1_/NCNC and Control Samples

**Figure 1:**
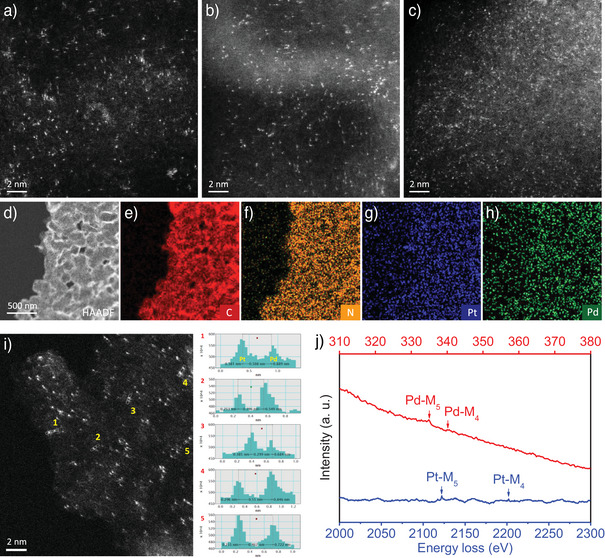
Electron microscopy characterizations. a) HAADF‐STEM image of Pt_1_Pd_1_/NCNC. b) HAADF‐STEM image of Pt_1_/NCNC. c) HAADF‐STEM image of Pd_1_/NCNC. d)–h) EDS‐mapping images of Pt_1_Pd_1_/NCNC. i) Local HAADF‐STEM image of Pt_1_Pd_1_/NCNC and corresponding intensity profiles in areas 1 to 5. j) EELS spectra of the Pt and Pd single atoms.

NCNC supports fast electron transfer due to their high conductivity and electrolyte accessibility.^[^ ^25^ ^]^ In fact, the microstructure of the NCNC support prepared in this study features nano‐, meso‐, and macro‐pores, and has a high specific surface area of 1063.5 m^2^ g^‒1^ as well as N content of 8.93 at% (Figure S1). The presence of small nanopores (0.6 nm) combined with the high N content has been shown to anchor single atoms.^[^ ^25^ ^]^ Pt_1_Pd_1_/NCNC DASCs, with a combined Pt and Pd loading of 3.294 wt% (2.106 wt% for Pt and 1.188 wt% for Pd), were synthesized by an impregnation–adsorption method (see Methods). SACs of Pt_1_/NCNC with Pt loading of 3.25 wt% or Pd_1_/NCNC with Pd loading of 3.26 wt% were also prepared using an analogous procedure. Figure 1 shows morphological and compositional characterizations of the catalysts. As revealed by bright spots in the high‐angle annular dark‐field scanning transmission electron microscopy (HAADF‐STEM) image of Pt_1_Pd_1_/NCNC, isolated Pt and Pd atoms are evenly dispersed on the NCNC support (Figure 1a). No nanoparticles are visible in the transmission electron microscopy (TEM) images (Figure S2). The corresponding elemental maps demonstrate that Pt, Pd, N, and C are uniformly distributed across the entire sample (Figure 1d–h), suggesting the co‐existence of Pt and Pd in Pt_1_Pd_1_/NCNC. Moreover, the dizygotic Pt and Pd atoms have inter‐nuclear distances ranging from 0.2 to 0.6 nm and heteronuclear Pt and Pd atoms could be distinguished in the corresponding intensity profiles (Figure 1i and S3). We performed electron energy‐loss spectroscopy (EELS) corresponding to Figure S4 and observed M_4,5_ peaks of Pt and Pd (Figure 1j), indicating the coexistence of Pt and Pd atoms. These results suggest the formation of dizygotic Pt and Pd sites. In fact, Pt and Pd atoms are atomically dispersed on Pt_1_/NCNC and Pd_1_/NCNC, respectively, as shown in corresponding HAADF‐STEM images (Figure 1b,c). Due to the atomically dispersed Pt/Pd and extremely low Pt/Pd loading, Pt or Pd peaks cannot be detected by X‐ray diffraction (XRD) in Pt_1_Pd_1_/NCNC, Pt_1_/NCNC, and Pd_1_/NCNC (Figure S5).

**Figure 2:**
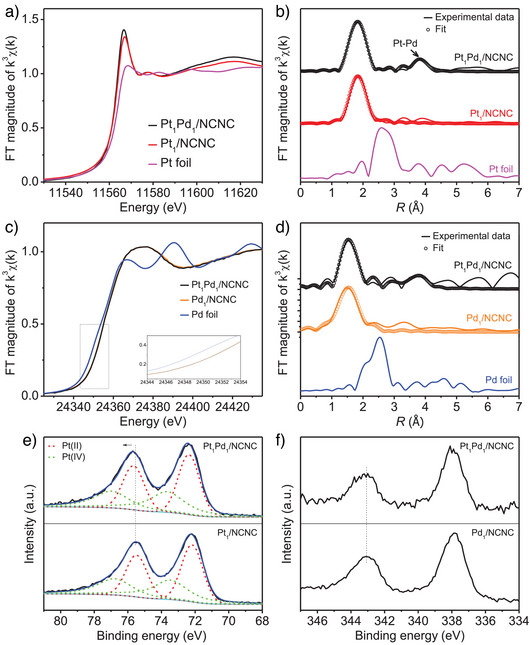
Structural characterization of Pt_1_/NCNC, Pd_1_/NCNC, and Pt_1_Pd_1_/NCNC by XAFS and XPS. a) Normalized XANES spectra at the Pt *L*_3_ edge. b) *k* ^3^‐weighted R‐space Fourier‐transformed spectra from EXAFS. c) Normalized XANES spectra at the Pd *K* edge. d) *k* ^3^‐weighted R‐space Fourier‐transformed spectra from EXAFS. e) XPS spectra for Pt 4*f*. f) XPS spectra for Pd 3*d*. In (a,b,c,d), the corresponding data for Pt or Pd foil are presented for comparison.

To further verify atomic dispersion and investigate the Pt and Pd coordination in the Pt_1_Pd_1_/NCNC and control samples, X‐ray absorption near‐edge structure (XANES), extended X‐ray absorption fine structure (EXAFS) spectrum, and X‐ray photoelectron spectroscopy (XPS) were performed (Figure 2). In the Pt *L*‐edge, the normalized XANES spectra of Pt_1_Pd_1_/NCNC and Pt_1_/NCNC have a stronger white‐line intensity at 11567 eV than that of Pt foil, indicating that Pt in Pt_1_Pd_1_/NCNC and Pt_1_/NCNC is positively charged (Figure 2a). It should be noted that the white line of Pt_1_/NCNC is slightly lower than that of Pt_1_Pd_1_/NCNC, indicating a lower oxidation state of Pt in Pt_1_/NCNC than in Pt_1_Pd_1_/NCNC.^[^ ^8^ ^]^ Figure 2b shows the *k* ^3^‐weighted Fourier transform EXAFS at Pt *L*‐edge. Compared to Pt foil, no peaks at 2.60 Å from Pt─Pt bond were detected in either Pt_1_Pd_1_/NCNC or Pt_1_/NCNC, indicating atomic dispersion Pt atoms in both catalysts, in agreement with the HAADF‐STEM micrographs. Instead, a peak around 1.83 Å corresponding to Pt─C/N bonds was detected in both Pt_1_Pd_1_/NCNC or Pt_1_/NCNC samples (Figure 2b).^[^ ^25^ ^]^ Unlike Pt_1_/NCNC, Pt_1_Pd_1_/NCNC exhibited a peak at approximately 3.82 Å, indicating the presence of a Pt···Pd heteronuclear atom site. This aligns with the HAADF‐STEM results, which revealed an average distance of 4.24 Å between Pt and Pd atoms (Figure 1i).^[^ ^26^ ^]^ Examining the normalized XANES spectra of Pd (Figure 2c), Pd_1_/NCNC showed a slightly higher oxidization state than Pt_1_Pd_1_/NCNC as evidenced by a corresponding shift in the XANES edge to higher energy.^[^ ^27^ ^]^ This higher oxidation state indicates that Pd atoms in the Pt_1_Pd_1_/NCNC are less positively charged compared to the Pd atoms in Pd_1_/NCNC. To further determine the configuration of Pt_1_Pd_1_/NCNC and Pt_1_/NCNC, the Fourier‐transformed EXAFS experimental spectra were fitted with DFT (see DFT calculations in the Methods section for more details). The EXAFS fitting results suggest that Pt in Pt_1_Pd_1_/NCNC has three coordinating interactions attributable to Pt─C (2.85 Å), Pt─N (2.17 Å), and Pt···Pd (4.10 Å) bonds, with corresponding coordination numbers 2, 2, and 1.1 (Table S1). Pd in Pt_1_Pd_1_/NCNC has peaks attributable to Pd─C/N (1.54 Å) and Pd─Pt (around 3.84 Å) bonds^[^ ^21^ ^]^ (Figure 2d, Table S2). Peaks at 2.51 Å corresponding to the Pd─Pd bond were absent in either Pt_1_Pd_1_/NCNC or Pd_1_/NCNC, indicating atomic dispersion of Pd atoms in both catalysts (Figure 2d). In addition, the wavelet‐transform results of Pt_1_Pd_1_/NCNC and Pt_1_/NCNC show a maximum intensity at ∼7.0 Å^−1^ corresponding to Pt─N/C bonds (Figure S6). In contrast to Pt_1_/NCNC, Pt_1_Pd_1_/NCNC shows an extra signal (marked by a square) (Figure S6a), which is likely derived from the Pt···Pd contribution.^[^ ^10^ ^]^

The Pt 4*f* XPS spectra show two peaks corresponding to Pt 4*f*_5/2_ and Pt 4*f*_7/2_ (Figure 2c). The binding energies of the Pt 4*f* were 72.55 and 75.85 eV for Pt_1_Pd_1_/NCNC, and 72.30 and 75.60 eV for Pt_1_/NCNC. Pt_1_Pd_1_/NCNC had a higher Pt 4*f* binding energy than Pt_1_/NCNC, indicating that Pt in Pt_1_Pd_1_/NCNC had a higher oxidation state, consistent with the XANES results. However, an analysis of the Pd 3*d* peak in Pt_1_Pd_1_/NCNC and Pd_1_/NCNC indicates that Pd has similar oxidation states in both materials, possibly resulting from the relatively low resolution of XPS. These results demonstrate that the coexistence of single‐atom Pd and Pt significantly influences their respective electronic structures.^[^ ^10^, ^21^ ^]^ The XPS peak of pyridinic N in Pt_1_Pd_1_/NCNC, Pt_1_/NCNC, and Pd_1_/NCNC slightly widened and shifted toward higher binding energies compared to pristine NCNC (Figure S7), suggesting that N interacts with atomically dispersed Pt and Pd,^[^ ^25^ ^]^ in agreement with the EXAFS coordination results and corresponding model analysis.

The above data indicate that Pt and Pd are atomically dispersed in Pt_1_/NCNC, Pd_1_/NCNC, and Pt_1_Pd_1_/NCNC. In addition, combined HAADF‐STEM, EXAFS, and XPS characterization and first‐principles modeling show that heteronuclear, atomically‐dispersed Pt and Pd sites are present in Pt_1_Pd_1_/NCNC.

### Electrocatalytic Activity

**Figure 3:**
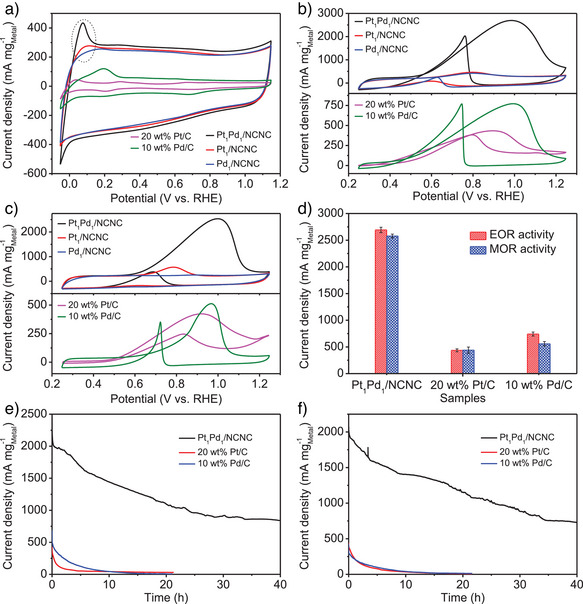
Electrochemical EOR and MOR performance of Pt_1_Pd_1_/NCNC and control samples. a) Representative CV curves in 0.1 mol L^‒1^ KOH. b) Representative CV curves in a 0.1 mol L^‒1^ KOH and 1 mol L^‒1^ ethanol aqueous solution. c) Representative CV curves in a 0.1 mol L^‒1^ KOH and 1 mol L^‒1^ methanol aqueous solution. d) mass activities based on ICP‐OES results. Chronoamperometric curves were obtained at 0.9 V (vs. RHE) in a 0.1 mol L^‒1^ KOH and 1 mol L^‒1^ e) ethanol aqueous solution and f) methanol aqueous solution.

Following the morphological and structural characterization, the electrocatalytic activity of Pt_1_Pd_1_/NCNC, Pt_1_/NCNC, Pd_1_/NCNC, 20 wt% Pt/C, and 10 wt% Pd/C were evaluated to assess the impact of coexisting Pt and Pd single atoms on NCNC (Figure 3). These samples were first tested in N_2_‐saturated KOH (0.1 mol L^‒1^) by cyclic voltammetry (CV). According to the integrated hydrogen desorption charge as circled in the positive potential scan (‒0.05 to 0.25 V versus the reversible hydrogen electrode (RHE)) (Figure 3a), the normalized electrochemical surface area (ECSA_HUPD_) based on hydrogen underpotential deposition was obtained. The ECSA_HUPD_ of Pt_1_/NCNC and Pd_1_/NCNC were 10.7 m^2^ g^‒1^_Pt_ and 0.2 m^2^ g^‒1^_Pd_, respectively. Unexpectedly, the Pt_1_Pd_1_/NCNC catalyst delivered an enhanced ECSA normalized with respect to the total mass of Pt and Pd of 90.8 m^2^ g^‒1^_Pt+Pd_. This value is much larger than those of Pt_1_/NCNC, Pd_1_/NCNC, 20 wt% Pt/C (15.9 m^2^ g^‒1^_Pt_), and 10 wt% Pd/C (73.8 m^2^ g^‒1^_Pd_). The higher ECSA_HUPD_ of Pt_1_Pd_1_/NCNC likely results from improved hydrogen adsorption and desorption due to Pt/Pd interaction.^[^ ^28^, ^29^ ^]^ However, ECSA_HUPD_ may be influenced by Faradaic contributions from hydrogen evolution and oxidation currents. Therefore, we also calculated ECSA_CO_ based on CO stripping (Figure S8). The ECSA_CO_ values of Pt_1_Pd_1_/NCNC, Pt_1_/NCNC, Pd_1_/NCNC, and 20 wt% commercial Pt/C were estimated to be 117.2, 95.5, 110.3, and 40.3 m^2^ g^‒1^_Metal_, respectively. Notably, Pt_1_Pd_1_/NCNC exhibits the highest ECSA among the samples. As ECSA can be correlated to the density of available active sites,^[^ ^28^ ^]^ these results indicate the bonding of Pt and Pd single atoms in the Pt_1_Pd_1_/NCNC provides a greater number of active sites relative to Pt and Pd alone.

EOR performance of the catalysts was evaluated in an aqueous solution of N_2_‐saturated KOH (0.1 mol L^‒1^) and ethanol (1 mol L^‒1^) (Figure 3b). CVs of Pt_1_/NCNC and Pd_1_/NCNC showed negligible electro‐oxidation peaks, suggesting that both Pt_1_/NCNC and Pd_1_/NCNC are virtually inactive toward EOR (Figure 3b). Conversely, the CV curves of Pt_1_Pd_1_/NCNC had obvious peaks in both the forward and backward scans, corresponding to ethanol and intermediates oxidation, respectively. It is noteworthy that the pristine NCNC, although it has many intrinsic defect sites, shows no activity toward AOR (Figures S1 and S9). These results indicate that the coexistence of Pt and Pd atoms on NCNC would affect EOR activity. The mass activity normalized with respect to the total metal mass of Pt_1_Pd_1_/NCNC was 2692.5 ± 50.4 mA mg^‒1^_metal_, which is approximately 6.3 and 3.6 times higher than 20 wt% Pt/C (427.3 ± 27.3 mA mg^‒1^_Pt_) and 10 wt% Pd/C (741.8 ± 37.5 mA mg^‒1^_Pd_) (Figure 3d), respectively. The mass activity is also significantly larger than most state‐of‐the‐art catalysts (Table S3).

Durability was assessed^[^ ^30^ ^]^ by testing the samples chronoamperometrically for 10 h at 0 V (versus Ag/AgCl). Pt_1_Pd_1_/NCNC maintained a mass activity of 2588.4 mA mg^‒1^_Pt_ with a slight degradation of 4.0% during testing, while 20 wt% Pt/C decreased its mass activity by ∼17.4% (434.1 to 358.6 mA mg^‒1^_Pt_) (Figure S10). We also compared the long‐term durability of Pt_1_Pd_1_/NCNC against commercial Pt/C and Pd/C benchmarks, by recording *i*‐*t* curves (Figure 3e). The mass activity retention of Pt_1_Pd_1_/NCNC was still 44.7% (1098.1 mA mg^−1^_Metal_) after 20 h, and 34.2% (839.7 mA mg^−1^_Metal_) after 40 h. The decrease in activity may be due to the aggregation of Pt and Pd single atoms to nanoclusters/nanoparticles (Figure S11). However, the benchmark Pt/C retained only 9.9% (42.5 mA mg^‒1^_Pt_) and 7.2% (31.2 mA mg^‒1^_Pt_) of its EOR mass activity after 10 and 20 h, respectively. Furthermore, Pd/C retained only 6.3% (45.1 mA mg‒1 Pd) and 1.1% (7.5 mA mg^‒1^_Pd_) of EOR mass activities after 10 and 20 h, respectively. These results indicate that Pt_1_Pd_1_/NCNC has better durability than Pt/C and Pd/C, also much better than that reported stability in literatures (Table S3).

Pt_1_/NCNC and Pd_1_/NCNC were shown to be inert toward MOR in an aqueous solution containing KOH (0.1 mol L^‒1^) and methanol (1 mol L^‒1^) (Figure 3c). Instead, Pt_1_Pd_1_/NCNC catalyst showed a MOR mass activity of 2578.6 ± 35.8 mA mg^‒1^_Metal_, which is 6.1 and 4.6 times higher than that of 20 wt% Pt/C (424.8 ± 35.6 mA mg^‒1^_Pt_) and 10 wt% Pd/C (558.9 ± 41.6 mA mg^‒1^_Pd_), respectively (Figure 3c,d). Furthermore, Pt/C retained only 7.6% (33.5 mA mg^‒1^_Pt_) and 2.1% (9.4 mA mg^‒1^_Pt_) of MOR mass activities after 10 and 20 h (Figure 3f), respectively. Pd/C retained only 9.3% (39.7 mA mg‒1 Pd) and 3.3% (14.0 mA mg^‒1^_Pd_) of MOR mass activities after 10 and 20 h, respectively. Instead, 54.3% (1152.5 mA mg^‒1^_Metal_) of the mass activity of Pt_1_Pd_1_/NCNC was still retained after 20 h, and 34.5% (729.2 mA mg^‒1^_Metal_) after 40 h, suggesting longer durability for Pt_1_Pd_1_/NCNC compared to Pt/C.

Additionally, Pt_1_Pd_1_/NCNC demonstrates turnover frequency (TOF) values of 2.73 ± 0.05 s^−1^ for EOR and 1.75 ± 0.02 s^−1^ for MOR, which are superior to those of commercial Pt/C (TOF values of 0.42 ± 0.03 s^−1^ for EOR and 0.84 ± 0.07 s^−1^ for MOR). These results indicate that dizygotic Pt and Pd atoms likely contribute to the greater performance toward EOR and MOR.

### AOR Mechanism

**Figure 4:**
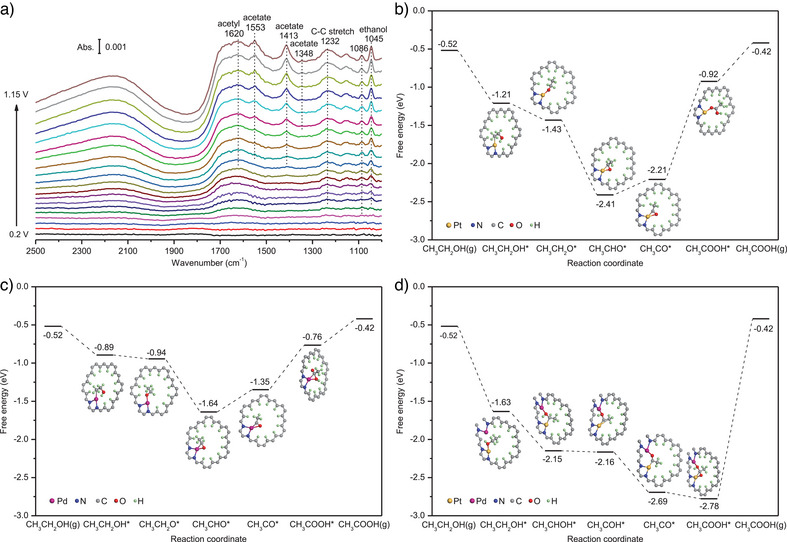
EOR mechanism. a) *Operando* IRRAS recorded during the EOR on Pt_1_Pd_1_/NCNC. b‐d) The electro‐oxidation of ethanol on b) Pt_1_/NCNC following pathway (1): CH_3_CH_2_OH→CH_3_CH_2_O→CH_3_CHO→CH_3_CO→CH_3_COOH. c) Pd_1_/NCNC following pathway (1): CH_3_CH_2_OH→CH_3_CH_2_O→CH_3_CHO→CH_3_CO→CH_3_COOH. d) Pt_1_Pd_1_/NCNC following pathway (2): CH_3_CH_2_OH→CH_3_CHOH→CH_3_COH→CH_3_CO→CH_3_COOH. Note: To avoid visualizing overlapping atoms, some structures in Figure 4b–d were rotated, thus leading to the width/height variations.

In order to gain insights into reaction mechanisms, *operando* IRRAS and DFT simulations were carried out (Figure 4). The spectra of ethanol oxidation on Pt_1_Pd_1_/NCNC were recorded in real‐time by sweeping the potential from 0.2 to 1.15 V versus RHE. As shown in Figure 4a, the characteristic bands at ∼1087 and ∼1045 cm^−1^ can be assigned to the C─O stretching vibration of ethanol, indicating the ethanol adsorption on the surface of the catalyst.^[^ ^31^, ^32^ ^]^ Absorption bands located at 1348, 1413, and 1553 cm^−1^ were also detected. These can be attributed to the bending vibration of −CH_3_ of adsorbed CH_3_COO^−^, as well as the symmetric and asymmetric stretching bands of O─C─O of CH_3_COO^−^, respectively.^[^ ^31^, ^33^ ^]^ The peaks at around 1620 and 1232 cm^‒1^ are assigned to vibration of C═O of adsorbed acetyl (CH_3_CO) and C─C stretch,^[^ ^34^ ^]^ respectively. It should be noted that adsorbed CO (1958–1991 cm^−1^) was absent during EOR. Online gas chromatography also revealed that neither CO nor CO_2_ were produced during the EOR on Pt_1_Pd_1_/NCNC (Figure S12). These results indicate that the C1 pathway was not significant. Therefore, EOR in Pt_1_Pd_1_/NCNC occurs through a C2 pathway. Thanks to the absence of poisonous CO during EOR, Pt_1_Pd_1_/NCNC shows higher stability than commercial Pt/C and Pd/C (see Figure 3e,f). Moreover, the liquid products of EOR were measured by high‐performance liquid chromatography (HPLC) (Figure S13) and ^1^H nuclear magnetic resonance (^1^H‐NMR) spectroscopy (Figure S14). These experiments showed only acetate formed during EOR. In other words, ethanol is electro‐oxidized to acetate as the final product in an alkaline solution. From ^1^H‐NMR characterization, the FEs for the ethanol‐to‐acetate reaction were estimated to be 94.6%, 97.5%, 95.0%, and 93.9%, at 0.85, 0.95, 1.05, and 1.15 V (versus RHE) (Figure S14b), respectively.

We conducted DFT calculations to understand this mechanism and the origin of enhanced EOR activities on Pt_1_Pd_1_/NCNC. Different models varying the distance between Pt and Pd were constructed. The structure optimized against the EXAFS data (Pt···Pd distance of 4.10 Å) was utilized to simulate the Pt_1_Pd_1_/NCNC (Figure S15a). In addition, we optimized two alternative configurations with nitrogen atoms placed differently (Figure S16) in comparison to the one used for electrocatalysis (Figure S15a). Both showed marginally higher total energies (0.957 and 1.203 eV above the chosen model). These findings further validate that the specific coordination environment in our suggested structure enhances thermodynamic stabilization for atomically dispersed Pt and Pd. In this regard, Pt_1_Pd_1_/NCNC, Pt_1_/NCNC, and Pd_1_/NCNC were also simulated (Figure S15).

To be consistent with IRRAS results, only the partial EOR toward acetate production was computed. The four different reaction pathways^[^ ^35^ ^]^ for oxidizing ethanol to acetate are shown in Figure S17. The reaction free energy diagrams were calculated following the literature (Table S4).^[^ ^8^, ^36^ ^]^ The vibrational frequencies of CH_3_CH_2_OH, CH_3_CH_2_O, CH_3_CHO, CH_3_CHOH, CH_3_CO, CH_3_COH, and CH_3_COOH adsorbed on Pt_1_Pd_1_/NCNC Pt_1_/NCNC and Pd_1_/NCNC are listed in Tables S5–S11. To evaluate the preference of different reaction mechanisms, we further calculated the onset potential, namely the maximum energy required to overcome the barrier of each elementary step. For Pt_1_/NCNC, all four reaction pathways (Figure S18) require an onset potential of 1.28 V, which is limited by the last step of CH_3_CO* → CH_3_COOH*. Figure 4b shows the diagram of free energies for the reaction pathway (1) proceeding as CH_3_CH_2_OH→CH_3_CH_2_O→CH_3_CHO→CH_3_CO→CH_3_COOH. For Pd_1_/NCNC, pathways (1) and (3) require an onset potential of 0.58 V to overcome the step of CH_3_CO*→CH_3_COOH* (Figures 4c and S19). Conversely, pathways (2) and (4) require an onset potential of 0.73 V due to the free energy difference between CH_3_CHOH* and CH_3_COH* (Figure S19). In contrast, the free energies of pathway (2) on Pt_1_Pd_1_/NCNC are reduced with no endothermic reaction steps (Figure 4d), implying that catalysis of ethanol toward acetic acid can take place spontaneously on Pt_1_Pd_1_/NCNC. For pathways (1), (3), and (4) on Pt_1_Pd_1_/NCNC, the onset potential values are 1.07, 1.07, and 1.00 V, respectively (Figure S20). In summary, EOR on Pt_1_/NCNC and Pd_1_/NCNC requires a *U*_onset_ of 1.28 and 0.58 V, respectively, while on Pt_1_Pd_1_/NCNC pathway (2) can take place with no onset potential required. This also agrees with the above experiments that show a faster EOR kinetics on Pt_1_Pd_1_/NCNC compared to Pt_1_/NCNC and Pd_1_/NCNC. The DFT results are consistent with IRRAS results that show acetate was the final oxidative product during EOR.

To understand how the combination of Pt and Pd lowers the acetate formation barrier, we carried out a principal interacting orbitals (PIOs) analysis^[^ ^37^, ^38^ ^]^ on the same model system studied above with a single adsorbed CH_3_CO. The PIO analysis can be used to identify the dominant interacting orbitals that are semi‐localized. Furthermore, it provides an easily interpretable bond index that can be linked to the interaction strength.^[^ ^37^, ^38^ ^]^ It should be remarked that we chose a CH_3_CO intermediate because the fundamental reaction step leading to CH_3_CO* formation is exothermic on Pt_1_Pd_1_/NCNC, while the same process is endothermic on Pt_1_/NCNC and Pd_1_/NCNC. We therefore expect that the PIO analysis will help elucidate the underpinning mechanisms that led to this difference. The calculated PIO‐based total bond index values between CH_3_CO and Pt_1_/NCNC, Pd_1_/NCNC, and Pt_1_Pd_1_/NCNC substrates were 1.76, 1.55, and 1.96, respectively. According to the PIO theory, the higher the calculated bond index, the stronger the interaction. Consequently, the stronger interaction between CH_3_CO and the Pt···Pd‐containing model implies lower adsorption energy than Pt_1_/NCNC and Pd_1_/NCNC. The calculated PIOs with the first three major contributions are shown in Figures S21–S23. For the Pt_1_Pd_1_/NCNC substrate, the carbon and oxygen in CH_3_CO interact with Pt and Pd to form one strong Pt‒C covalent bond (with a PIO‐based bond index of 0.914) and one Pd─O coordinate bond (with PIO‐based bond index of 0.430), respectively, thus resulting in stronger total interaction between CH_3_CO and Pt···Pd center. Indeed, the C═O bond in the Pt_1_Pd_1_/NCNC is the strongest among all three substrates. In Pd_1_/NCNC or Pt_1_/NCNC substrate, both carbon and oxygen atoms coordinate with one metal, resulting in the severe distortion of the adsorbed CH_3_CO. The carbon in CH_3_CO is *sp* ^2^ hybridized, whereas the bond angle of carbon deviates farther from 120 degrees. Consequently, the orbital overlap in such a distorted CH_3_CO molecule is diminished, and the PIO‐based bond indices in Pd_1_/NCNC or Pt_1_/NCNC are also smaller in comparison to that of Pt_1_Pd_1_/NCNC.

We also analyzed the d‐band center for the Pt_1_/NCNC, Pd_1_/NCNC, and Pt_1_Pd_1_/NCNC systems. As shown in Table S12, the d‐band center of Pt in Pt_1_Pd_1_/NCNC shifts upward toward the Fermi level compared to Pt_1_/NCNC, while the d‐band center of Pd moves slightly downward relative to Pd_1_/NCNC. Based on the d‐band model developed by Nørskov and colleagues^[^ ^39^, ^40^ ^]^ the upward shift of Pt increases the adsorption strength of intermediates such as CH_3_CH_2_OH (refer to Figure 4), whereas the downward shift of Pd reduces the adsorption of species like CH_3_CHO (see Figures S19 and S20). This synergistic adjustment of adsorption energies at the heteronuclear Pt–Pd sites promotes intermediate conversion and effectively lowers the energy barrier for ethanol oxidation.

**Figure 5:**
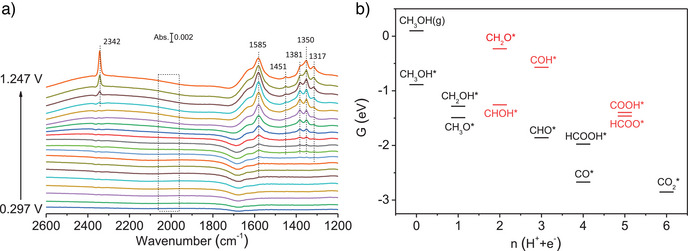
MOR mechanism. a) *Operando* IRRAS recorded during the MOR on Pt_1_Pd_1_/NCNC. b) The calculated free energies of all possible intermediates on the Pt_1_Pd_1_/NCNC system. The black color denotes intermediates formed from preceding ones accompanied by decreased free energy. The red color indicates intermediates that might be absent in the reaction, as additional energy is needed to surpass the reaction barrier. The *x*‐axis represents the number of proton/electron pairs produced from the initial reactants.

To study the MOR mechanisms, *operando* IRRAS was carried out (Figure 5a). As for Pt_1_Pd_1_/NCNC, in situ IRRAS shows an absorption band at 2342 cm^−1^, corresponding to the asymmetric stretch vibration of CO_2_.^[^ ^41^ ^]^ In addition, three bands around 1381 and 1350/1317 cm^−1^ were also observed, corresponding to CO_3_^2−^ and HCO_3_^−^, respectively. These two species are sequentially generated from the reaction between CO_2_ and the electrolyte.^[^ ^42^ ^]^ Moreover, an absorption band around 1585 cm^−1^ was also observed, which can be attributed to HCOO^−^.^[^ ^43^ ^]^ However, it should be noted that triply bonded CO (CO_T_) (1802–1786 cm^−1^)^[^ ^33^ ^]^ and linearly bonded CO (CO_L_) (2060–1990 cm^−1^)^[^ ^44^, ^45^ ^]^ were not detected, indicating that methanol electro‐oxidation on Pt_1_Pd_1_/NCNC produces HCOO^−^ as an intermediate, CO_2_ as a final product, and no CO. In contrast, during MOR on 20 wt% commercial Pt/C, CO_L_ was also observed in addition to HCOO^−^, CO_2_, CO_3_^2−^, and HCO_3_^−^ (Figure S24). This implies that CO was one of the intermediates generated during MOR. Based on the above results, the MOR reaction on Pt_1_Pd_1_/NCNC is likely to occur through the following pathway: CH_3_OH→HCOOH→CO_2_.

To supplement the aforementioned analysis and understand the MOR mechanism on Pt_1_Pd_1_/NCNC, we conducted DFT calculations based on the MOR reaction diagram given in Figure S25. The vibrational frequencies of CH_3_OH, CH_2_OH, HCOOH, CH_3_O, CH_2_O, CHOH, COOH, HCOO, CHO, CO_2_, COH, and CO intermediates adsorbed on Pt_1_Pd_1_/NCNC are listed in Tables S13 and S14. As shown in Figure 5b, the adsorption of CH_3_OH (g) on the Pt_1_Pd_1_/NCNC system (CH_3_OH*) is energetically favorable, with a decrease of 0.98 eV. For the first elementary reaction, CH_3_OH*→CH_2_OH* and CH_3_OH*→CH_3_O* are exothermic. However, the reaction barrier of CH_3_O*→CH_2_O* (1.26 eV) is much higher than CH_2_OH*→CHOH* (0.03 eV), implying that CHOH* is the most likely product in the secondary step. Starting from CHOH*, CHOH*→CHO* can take place spontaneously, whereas CHOH*→COH* is endothermic, suggesting that the product of the third step is CHO*. Regarding the fourth step, both CHO*→HCOOH* and CHO*→CO* have Δ*G* < 0, suggesting they are both spontaneous. The subsequent step, CO*→COOH*, is endothermic, requiring an additional energy of 1.27 eV, whereas HCOOH*→HCOO* only needs 0.51 eV. This suggests that HCOO* is more likely to be observed than COOH* in experiments. The final product of CO_2_* can be formed from the elementary reaction HCOO*→CO_2_*, which is associated with an energy release of −1.39 eV. From the analysis above, we can conclude that the pathway CHO*→HCOOH*→HCOO*→CO_2_* (onset potential of 0.51 V) is more favorable than CHO*→CO*→COOH*→CO_2_* (onset potential of 1.27 V), further corroborating the experimental results.

Based on the above experiments and simulations, the presence of Pt–Pd dimers on NCNC favors EOR and MOR, unlike individual single atoms of Pt and Pd on NCNC, which are inert. Pt_1_Pd_1_/NCNC exhibits higher EOR and MOR mass activities compared to commercial Pt/C and Pd/C. As revealed by *operando* IRRAS and online GC, Pt_1_Pd_1_/NCNC electrooxidizes ethanol and methanol through a direct pathway that bypasses the production of CO intermediate. Conversely, EOR and MOR on commercial Pt/C proceed through an indirect pathway involving the production of CO intermediate. Hence, unlike commercial Pt/C, Pt_1_Pd_1_/NCNC is not subject to CO poisoning, leading to a longer EOR and MOR stability.

## Conclusion

The DASCs of Pt_1_Pd_1_/NCNC and the SACs of Pt_1_/NCNC, and Pd_1_/NCNC were prepared using a simple impregnation–adsorption method. Pt_1_/NCNC and Pd_1_/NCNC are virtually inert toward alcohol oxidation, while Pt_1_Pd_1_/NCNC exhibited good ethanol and methanol electro‐oxidation with mass activities of 2692.5 ± 50.4 mA mg^‒1^_metal_ and 2578.6 ± 35.8 mA mg^‒1^_metal_, two values higher than those recorded for the commercial Pt/C and Pd/C benchmark catalysis. Combined *operando* IRRAS, online GC, HPLC, and NMR revealed that the EOR on Pt_1_Pd_1_/NCNC mainly proceeded through a C2 pathway with acetate ion as the final product. MOR on Pt_1_Pd_1_/NCNC had CO_2_ as the final product. Both EOR and MOR on Pt_1_Pd_1_/NCNC proceeded through a direct pathway without the generation of CO as an intermediate. This results in greater long‐term stability for Pt_1_Pd_1_/NCNC compared to the commercial benchmarks of Pt/C and Pd/C. Through the C2 pathway, Pt_1_Pd_1_/NCNC required no onset potential via a spontaneous process as calculated by first‐principles simulations. In contrast, Pt_1_/NCNC and Pd_1_/NCNC required an onset potential of 1.28  and 0.58 V, respectively. Moreover, EOR on Pt_1_/NCNC and Pd_1_/NCNC was limited by the CH_3_CO* to the CH_3_COOH* step. The exceptional performance of Pt_1_Pd_1_/NCNC DASCs not only demonstrates their potential for alcohol oxidation reactions but also opens up new avenues for designing highly efficient and stable electrocatalysts.

## Conflict of Interests

The authors declare no conflict of interest.

## Supporting information



Supporting Information

## Data Availability

The data that support the findings of this study are available from the corresponding author upon reasonable request.
