# Supplementary material for: Dizygotic Atomic Platinum and Palladium on Carbon for High‐Performance Ethanol and Methanol Electro‐Oxidation
Source: Angew Chem Int Ed Engl. 2025 Jul 22;64(37):e202502348. doi: 10.1002/anie.202502348 (PMC12416477; doi:10.1002/anie.202502348)
Supplement: Supplementary file 1 — Supporting Information [file ANIE-64-e202502348-s001.docx]

Supplementary Information

**Dizygotic atomic platinum and palladium on carbon for high-performance ethanol and methanol electro-oxidation**

Zhiqi Zhang,*^,+^ Jiapeng Liu,^+^ Shangqian Zhu,^+^ Yuhao Wang^+^ Jian Wang, Minhong Xu, Jie Zhao, Zheng Wang, Dewang Zeng, Jianrong Zeng, Yufei Song, Chih-Wen Pao, Zhiwei Hu, Jongwoo Lim, Rui Xiao, Minhua Shao, and Francesco Ciucci*

**EXPERIMENTAL SECTION**

**Materials.** H_2_PtCl_6_·6H_2_O, PdCl_2_, Commercial Pt/C (20 wt% Pt), and Pd/C (10 wt% Pd) were purchased from Sigma-Aldrich. Ethanol and methanol were purchased from Scharlab.

**Synthesis of Pt_1_Pd_1_/NCNC, Pt_1_/NCNC, and Pd_1_/NCNC catalysts.** The NCNC support was prepared through an *in situ* MgO template method with pyridine precursors.[^1^](#_ENREF_1) The Pt_1_Pd_1_/NCNC catalysts were synthesized using an impregnation-adsorption method. 50 mg of NCNC was dispersed in 80 mL of deionized water, and then 3 mL of mixed solution (H_2_PtCl_6_·6H_2_O (0.002 mol L^‒1^) and PdCl_2_ (0.002 mol L^‒1^)) was added dropwise. After stirring at 70 °C for 5 h, the suspension was filtered. Then, the collected powder was washed with water and ethanol and dried in an oven at 70 °C for 2 h. Pt_1_/NCNC and Pd_1_/NCNC were prepared in a similar way by adding either an H_2_PtCl_6_·6H_2_O or a PdCl_2_ solution into the NCNC suspension, respectively. The Pt_1_Pd_1_/NCNC-500 catalyst was obtained by treating Pt_1_Pd_1_/NCNC at 500 ^o^C for 1 h under N_2_ flow.

**Catalyst characterization.** The morphology and structure of the catalysts were characterized by XRD (PANalytical X’pert Pro, Cu Kα radiation), TEM (JEOL-2010F), and aberration-corrected STEM (JEOL JEM-ARM200F). The chemical states of the catalysts were measured by XPS (Axis Ultra DLD). XAFS characterization was conducted at the 10C beamline of the Pohang Light Source (PLS) in the Pohang Accelerator Laboratory (PAL, Republic of Korea), at the BL13SSW beamline of the Shanghai Synchrotron Radiation Facility (SSRF, China), and at the TPS 44A beamline of National Synchrotron Radiation Research Center. The Pt *L*_3_-edge and Pd *K*-edge spectra were collected in fluorescent mode and were processed and analyzed using the ATHENA and ARTEMIS packages.[^2^](#_ENREF_2) For the fitting of Pt, the R and K values are the ranges of 1.5 to 4.7, and 3.0 to 11, respectively. For Pd, the R and K values are in the ranges of 1.0 to 4.5, and 3.0 to 10.7, respectively. The specific surface area and pore structure of NCNC were measured on a Thermo Fisher Scientific Surfer Gas Adsorption Porosimeter at 77 K. The gaseous products were analyzed by online gas chromatography (GC, hope-9860-5CNJ). The ^1^H-NMR was performed on a BRUKER AVANCE III 400 MHz. To determine the liquid products through ^1^H-NMR characterization, 1 mL of the post-reaction solution was combined with 0.1 mL dimethyl sulfoxide internal standard. The Pt and Pd content in these catalysts were analyzed by inductively coupled plasma-optical emission spectrometer (ICP-OES, PerkinElmer Avio 500).

The carbon balance from ethanol to acetate was calculated according to 4-electron pathways in alkaline solution. The 4-electron pathway is as follows:

$$\mathrm{CH}_{3}\mathrm{CH}_{2}OH+5\mathrm{OH}^{-}\to\mathrm{CH}_{3}\mathrm{COO}^{-}+4H_{2}O+4e^{-}$$

The faradaic efficiency (FE) of ethanol to acetate was calculated as follows:

$$\mathrm{FE}\left( \% \right)=\frac{znF}{q}\times100\%$$

where *z* is the theoretical number of electrons exchanged to form the desired product, *n* is the number of moles produced, F is the Faradaic constant (96485 C mol^−1^), and *q* is the total charge supplied during the process.

**Electrochemical characterization.** Electrochemical tests were conducted at 25 °C using a three-electrode setup and a CHI 900D workstation (CH Instruments). An Ag/AgCl (saturated KCl) and a graphite rod electrode were selected as the reference and counter electrode, respectively. The working electrode was prepared as follows: i) 1 mg of catalyst was ultrasonically dispersed in a mixed solution of 200 μL of water, 50 μL of ethanol, and 20 μL of Nafion (Dupont, 5 wt%); ii) 5 μL of the above ink was dropped onto a glassy-carbon electrode (Pine Instrument Co, 4 mm in diameter), followed by drying at room temperature. The electrochemical surface areas (ECSAs) of the catalysts were obtained via the hydrogen adsorption/desorption region in 0.1 mol L^−1^ KOH solution in cyclic voltammograms. The electrocatalytic activity towards MOR/EOR was measured by CV in an N_2_-saturated aqueous solution containing 0.1 mol L^−1^ KOH and 1 mol L^−1^ methanol/ethanol. CV scans were performed at a rate of 50 mV s^−1^ until a consistent response was recorded. In the CO stripping measurements, a monolayer of CO was adsorbed on the catalyst by flowing CO in 0.1 mol L^−1^ KOH for 20 min while holding the electrode potential at −0.95 V (vs. Ag/AgCl). Nonadsorbed CO was removed by bubbling the electrolyte with N_2_ for 20 min. Stripping measurements were initiated from −0.95 to 0 V (vs. Ag/AgCl) in the forward scan at 50 mV s^−1^ for at least two consecutive scans. All recorded potentials were converted to the reversible hydrogen electrode (RHE) using the formula *E*_RHE_ = *E*_Ag/AgCl_ + 0.198 + 0.059 × pH.

TOF was obtained by the following equations:[^3-5^](#_ENREF_3)

$$\mathrm{SD}\left[ \mathrm{site}g^{-1} \right]=\frac{Q_{\mathrm{co}}[Cg^{-1}]\times N_{A}[\mathrm{mol}^{-1}]}{n_{strip}\times F[C\mathrm{mol}^{-1}]}$$

$$Q_{\mathrm{co}} \left[ C g^{-1} \right]=\frac{area[AV]}{scan rate \left[ Vs^{-1} \right]\times m[g]}$$

$$TOF \left[ s^{-1} \right]=\frac{j[A g^{-1}]\times N_{A}[\mathrm{mol}^{-1}]}{nF[A s\mathrm{mol}^{-1}]\times SD \left[ \mathrm{site}g^{-1} \right]}$$

where F and $N_{A}$ are Faraday’s and Avogadro’s constants, respectively, $Q_{\mathrm{co}}$ is the CO stripping charge, $\mathrm{SD}$ is the site density, and $n_{strip}$ is the number of electrons involved in the oxidation of one CO molecule (here $n_{strip}=$ 2). The area was estimated by CO stripping conducted at a scan rate of 50 mV s^-1^. The parameter m is the mass of Pt and/or Pd on glassy carbon electrode based on the ICP results. The quantity $n$ represents the number of electrons transferred for each molecule of product generated. In the case of EOR on Pt₁Pd₁/NCNC, the end product is identified as acetic acid through IRRAS analysis, which corresponds to $n$ = 4. Conversely, EOR on 20 wt% commercial Pt/C results in CO₂ as the final product, with $n$ = 12. For MOR, both Pt₁Pd₁/NCNC and the 20 wt% commercial Pt/C also yield CO₂ as the final product, each having $n$ = 6.

***In Situ* Infrared Absorption Studies.** A Nicolet iS50 Fourier transform infrared (FTIR) spectrometer equipped with a mercury cadmium telluride (MCT) detector cooled with liquid nitrogen was used for the *operando* spectroelectrochemical FTIR studies. All spectra are displayed in terms of absorbance, quantified as [‒log(R/R_0_)], where R and R_0_ correspond to the spectra of the sample and reference, respectively. Spectra were collected continuously during the potential sweep from low to high potentials with a scanning rate of 5 mV s^‒1^. The resolution of the real-time FTIR reflection absorption spectroscopy spectra was 8 cm^‒1^ with 44 scans per spectrum and a collection duration of each 10 s.

**DFT calculations.** All spin-polarized first-principle calculations were performed using the Vienna ab *initio* simulation package (VASP)[^6^](#_ENREF_6)^,^ [^7^](#_ENREF_7) with a plane-wave basis set. The energy cutoff was set to 480 eV. Projector augmented wave (PAW)[^8^](#_ENREF_8) pseudopotentials with valence-electron configurations of 1*s*^1^, 2*s*^2^2*p*^2^, 2*s*^2^2*p*^3^, 2*s*^2^2*p*^4^, 5*s*^1^4*d*^9^ and 6*s*^1^5*d*^9^ were employed for H, C, N, O, Pd, and Pt, respectively. The Perdew-Burk-Ernzerhof (PBE) functional[^9^](#_ENREF_9) under the generalized gradient approximation (GGA) scheme was used to model the electron exchange correlation. Following the previous study,[^10^](#_ENREF_10) we simulated the NCNC substrate using a modified 6×6 graphene sheet with a hole of 0.6 Å. A single Pt/Pd atom bonded to the fringe N and/or C atoms was constructed to simulate the Pt_1_/NCNC and Pd_1_/NCNC, as illustrated in Figure S15. To simulate the dizygotic Pt···Pd anchored on the NCNC, structures with different placements of Pt and Pd were initially constructed and optimized. Finally, the model matching most closely to the EXAFS data was used to simulate the Pt_1_Pd_1_/NCNC (Figure S15a). The distance between Pt and Pd in this structure measured 4.12 Å. A vacuum space of 20 Å along the *c* direction was added to avoid self-interactions between neighboring substrates. The atomic positions were fully relaxed using Γ-centered *k-*points of 3×3×1 until the energy and force converged within 10^‒5^ eV and 0.02 eV/Å, respectively. The zero damping DFT-D3 method[^11^](#_ENREF_11) was also used to correct for van der Waals interactions.

All free energies were calculated relative to H_2_O, CO_2_, and H_2_ following the literature.[^12^](#_ENREF_12) For example, the free energy of ethanol was calculated following the reaction:

| $2\text{C}\text{O}_{\text{2}}\left( g \right)+6\text{H}_{\text{2}}\left( g \right)\rightleftharpoons\text{C}\text{H}_{\text{3}}\text{C}\text{H}_{\text{2}}\text{OH}\left( g \right)+3\text{H}_{\text{2}}\text{O}\left( l \right)$ | (1) |
| --- | --- |

and its free energy was calculated using

| $\Delta G\left( \text{C}\text{H}_{\text{3}}\text{C}\text{H}_{\text{2}}\text{OH} \right)=\Delta E+\Delta ZPE-T\Delta S=\left( E\left( \text{C}\text{H}_{\text{3}}\text{C}\text{H}_{\text{2}}\text{OH} \right)+3\times E\left( \text{H}_{\text{2}}\text{O} \right)-2\times E\left( \text{C}\text{O}_{\text{2}} \right)-6\times E\left( \text{H}_{\text{2}} \right) \right)+\left( ZPE\left( \text{C}\text{H}_{\text{3}}\text{C}\text{H}_{\text{2}}\text{OH} \right)+3\times ZPE\left( \text{H}_{\text{2}}\text{O} \right)-2\times ZPE\left( \text{C}\text{O}_{\text{2}} \right)-6\times ZPE\left( \text{H}_{\text{2}} \right) \right)-T\times\left( S\left( \text{C}\text{H}_{\text{3}}\text{C}\text{H}_{\text{2}}\text{OH} \right)+3\times S\left( \text{H}_{\text{2}}\text{O} \right)-2\times S\left( \text{C}\text{O}_{\text{2}} \right)-6\times S\left( \text{H}_{\text{2}} \right) \right)$ | (2) |
| --- | --- |

where $\Delta E$ is the difference of DFT energies, $\Delta ZPE$ corresponds to the zero-point energy, and $\Delta S$ denotes the entropy difference, $T$  is the standard temperature (298 K).

Free energies of intermediate adsorbates were calculated following a similar procedure. For example, the free energy of CH_3_CHO* was calculated following the reaction

| $2\text{C}\text{O}_{\text{2}}\left( g \right)+5\text{H}_{\text{2}}\left( g \right)+*\leftrightharpoons{\text{C}\text{H}_{\text{3}}\text{CHO}}^{\text{*}}+3\text{H}_{\text{2}}\text{O}\left( l \right)$ | (3) |
| --- | --- |

and its free energy change is given by

| $\Delta G\left( {\text{C}\text{H}_{\text{3}}\text{CHO}}^{\text{*}} \right)=\Delta E+\Delta ZPE-T\Delta S=\left( E\left( {\text{C}\text{H}_{\text{3}}\text{CHO}}^{\text{*}} \right)+3\times E\left( \text{H}_{\text{2}}\text{O} \right)-2\times E\left( \text{C}\text{O}_{\text{2}} \right)-5\times E\left( \text{H}_{\text{2}} \right)-E\left( \text{clean} \right) \right)+\left( ZPE\left( {\text{C}\text{H}_{\text{3}}\text{CHO}}^{\text{*}} \right)+3\times ZPE\left( \text{H}_{\text{2}}\text{O} \right)-2\times ZPE\left( \text{C}\text{O}_{\text{2}} \right)-5\times ZPE\left( \text{H}_{\text{2}} \right) \right)-T\times\left( S\left( {\text{C}\text{H}_{\text{3}}\text{CHO}}^{\text{*}} \right)+3\times S\left( \text{H}_{\text{2}}\text{O} \right)-2\times S\left( \text{C}\text{O}_{\text{2}} \right)-5\times S\left( \text{H}_{\text{2}} \right) \right)$ | (4) |
| --- | --- |

where $E\left( \text{clean} \right)$ is the energy of the pristine substrate, $E\left( {\text{C}\text{H}_{\text{3}}\text{CHO}}^{\text{*}} \right)$ is the energy of CH_3_CHO adsorbed on the substrate, $ZPE\left( {\text{C}\text{H}_{\text{3}}\text{CHO}}^{\text{*}} \right)$ and $S\left( {\text{C}\text{H}_{\text{3}}\text{CHO}}^{\text{*}} \right)$ are the zero-point energy correction and entropy of CH_3_CHO adsorbate, respectively. The zero-point energy and entropy values were all obtained using VASPKIT.[^13^](#_ENREF_13) All the intermediates were confirmed to be stable with no imaginary frequency.

To account for the electrochemical potential, we used the computational standard hydrogen electrode (SHE),[^12^](#_ENREF_12)^,^ [^14^](#_ENREF_14) where the change in free energy of a reaction involving the formation of a proton-electron pair (H^+^ + e^–^) versus that at the SHE was calculated as ∆*G*_U_ = – *eU*, where *U* is the applied potential. Hence, the onset potential for a given reaction pathway was calculated as

| $U_{\mathrm{onset}}=\max\left( \frac{{\Delta G}_{\mathrm{SHE}}^{i}}{e} \right)$ | (5) |
| --- | --- |

where *i* refers to the step number along a given pathway, ∆*G*_SHE_ is the calculated free energy change for the step at 0 V versus SHE. For a reaction with more than one possible pathway, e.g., the EOR in this work, the overall onset potential can be assessed as the minimum for all pathways. In the case of the MOR, the method used was similar to the one employed for EOR as the onset potential for the MOR was also determined using (5).

The PIOs analysis was conducted based on the checkpoint file generated by Gaussian 09.[^15^](#_ENREF_15) The single-point calculations were carried out at the PBE level[^9^](#_ENREF_9) with the 6-31G* basis set for C, O, H, and N atoms. The SDD effective core potentials[^16^](#_ENREF_16)^,^ [^17^](#_ENREF_17) were chosen for Pd and Pt atoms. For the PIO analysis, we retrieved the fragments with CH_3_CO adsorbed on the substrates from the DFT optimization and did not further optimize the structure in Gaussian 09. This ensures, to the most extent, the consistency of analysis from Gaussian and VASP. To passivate the dangling bond at the boundary C atoms, we introduced fictitious hydrogen atoms to saturate the boundary C atoms. Since we are mainly interested in the central metal atom and the intermediate, the added H atoms have negligible effect on the final results.


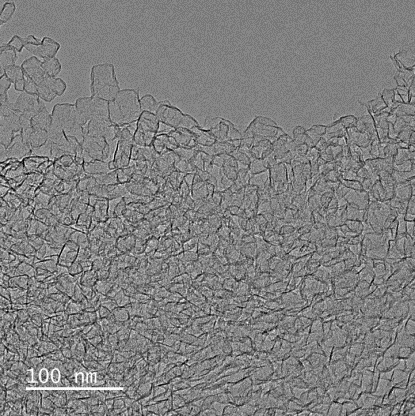

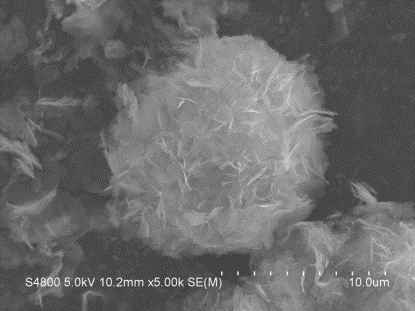

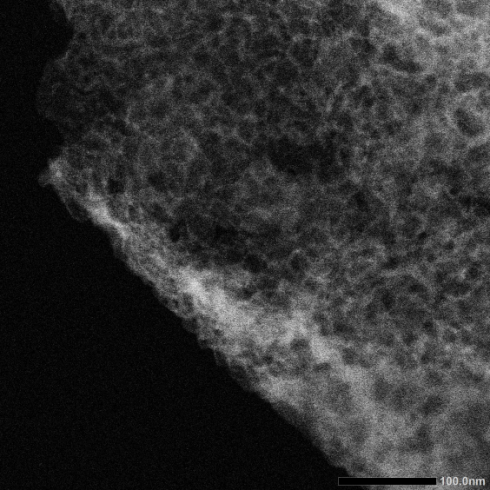


100 nm

100 nm

a

b

c

d

e

f

**Figure S1.** Characterizations of NCNC. (a) SEM image, (b) TEM image, (c) HAADF-STEM image, (d) N_2_ adsorption-desorption isotherm, (e) Pore size distributions, and (f) XPS survey scan.


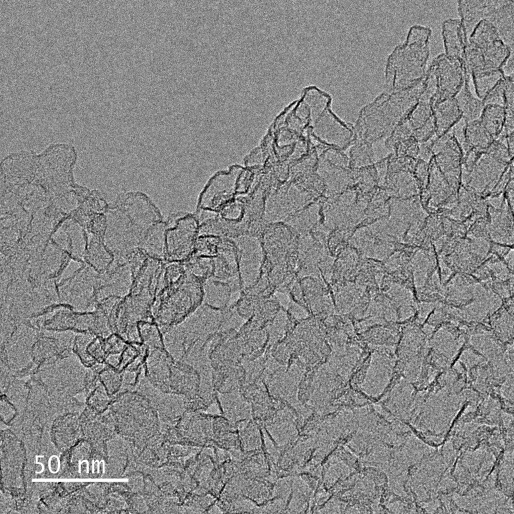

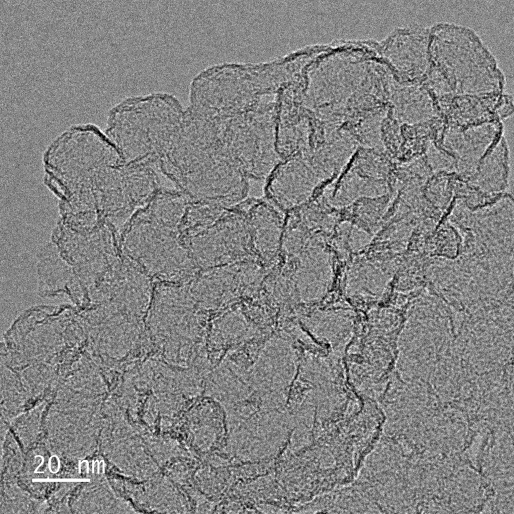

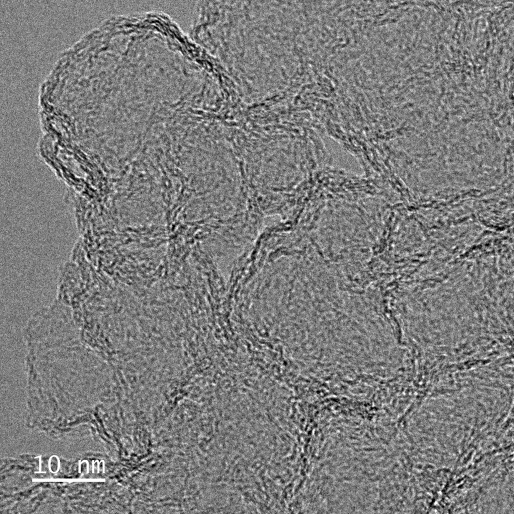

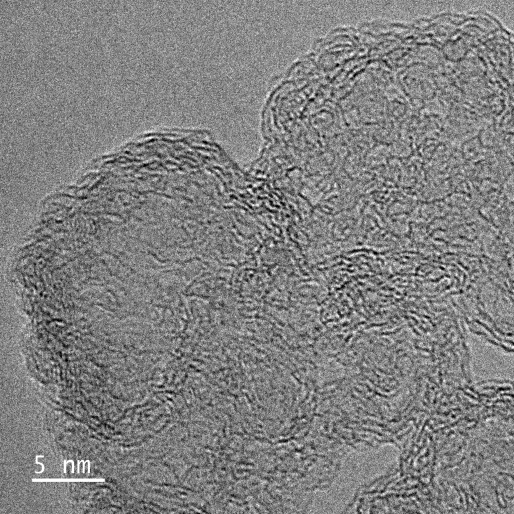


a

b

c

d

**Figure S2.** Typical (HR)TEM images of Pt_1_Pd_1_/NCNC.

**Figure S3.** The distance of the Pt and Pd dots was determined by counting 31 Pt-Pd pairs.


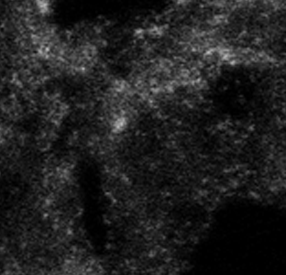


**2 nm**

**Figure S4.** HAADF-STEM image of Pt_1_Pd_1_/NCNC for EELS.

**Figure S5.** XRD patterns of Pt_1_Pd_1_/NCNC, Pt_1_/NCNC, Pd_1_/NCNC, and NCNC.

**Figure S6.** Wavelet transforms from experimental data. (a) Pt_1_Pd_1_/NCNC, (b) Pt_1_/NCNC.

**Figure S7.** N1s XPS spectra of Pt_1_Pd_1_/NCNC, Pd_1_/NCNC, Pt_1_/NCNC, and NCNC.

**Figure S8.** CO stripping tests in 0.1 mol L^‒1^ KOH at a scan rate of 50 mV s^‒1^. (a) Pt_1_/NCNC. (b) Pd_1_/NCNC. (c) Pt_1_Pd_1_/NCNC. (d) 20 wt% commercial Pt/C. The black and red curves correspond to the first and second scans, respectively. The first scan was recorded in the presence of CO adsorbed on the electrode, while the second scan was recorded in the absence of CO.

**Figure S9.** CV curves of pristine NCNC in (**a**) KOH (0.1 mol L^-1^), (**b**) 0.1 mol L^‒1^ KOH and 1 mol L^‒1^ ethanol, (**c**) 0.1 mol L^‒1^ KOH and 1 mol L^‒1^ methanol aqueous solution.

**Figure S10.** EOR performance before and after stability test. (a) Pt_1_Pd_1_/NCNC, (b) 20 wt% Pt/C.


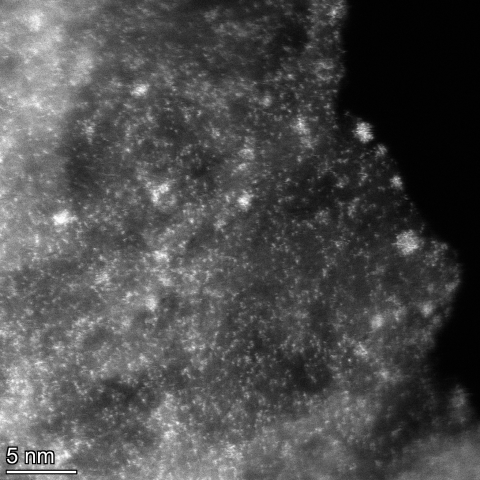


**Figure S11.** HAADF-STEM image of Pt_1_Pd_1_/NCNC after EOR stability test.

**Figure S12.** Online gas chromatography during EOR on Pt_1_Pd_1_/NCNC.

**Figure S13.** HPLC data of liquid product obtained after EOR on Pt_1_Pd_1_/NCNC. The peak (black line) with a retention time of around 11.16 min can be attributed to the existence of acetate as compared to acetate control (magenta line).

**Figure S14.** ^1^H-NMR spectroscopy at different potential ranges (a) and corresponding FE from ethanol to acetate (b) for Pt_1_Pd_1_/NCNC. The peaks with a chemical shift at 1.16 and 3.63 ppm were attributed to ethanol reactant in the electrolyte[^18^](#_ENREF_18). The chemical shift peaks at 2.70 and 4.79 ppm were ascribed to dimethyl sulfoxide (DMSO) internal standard and H_2_O, respectively[^19^](#_ENREF_19). The peak at 1.9 ppm was attributed to acetate (CH_3_COO^−^) produced during EOR[^20^](#_ENREF_20)^,^ [^21^](#_ENREF_21) Apart from these peaks, no other peaks were observed in the ^1^H-NMR spectroscopy.

a

b

c

Pt

Pd

N

C

H

4.12 Å

**Figure S15.** Optimized structures. (a) Pt_1_Pd_1_/NCNC, (b) Pt_1_/NCNC, and (c) Pd_1_/NCNC.

**Figure S16.** Typical structures optimized with N atoms placed at different sites in comparison to the one utilized in the manuscript.


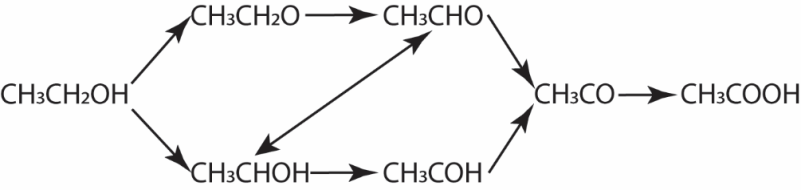


**Figure S17.** Reaction diagram for oxidation of ethanol to acetic acid, including four paths from ethanol to acetic acid:

Pathway (1): CH_3_CH_2_OH → CH_3_CH_2_O → CH_3_CHO → CH_3_CO → CH_3_COOH

Pathway (2): CH_3_CH_2_OH → CH_3_CHOH → CH_3_COH → CH_3_CO → CH_3_COOH

Pathway (3): CH_3_CH_2_OH → CH_3_CHOH → CH_3_CHO → CH_3_CO → CH_3_COOH

Pathway (4): CH_3_CH_2_OH → CH_3_CH_2_O → CH_3_CHO → CH_3_CHOH → CH_3_COH → CH_3_CO → CH_3_COOH

**Figure S18.** Electrochemical EOR on Pt_1_/NCNC following (a) pathway (2), (b) pathway (3), and (c) pathway (4).

**Figure S19.** Electrochemical EOR on Pd_1_/NCNC following: (a) pathway (2), (b) pathway (3), and (c) pathway (4).

**Figure S20.** Electrochemical EOR on Pt_1_Pd_1_/NCNC following: (a) pathway (1), (b) pathway (3), and (c) pathway (4).


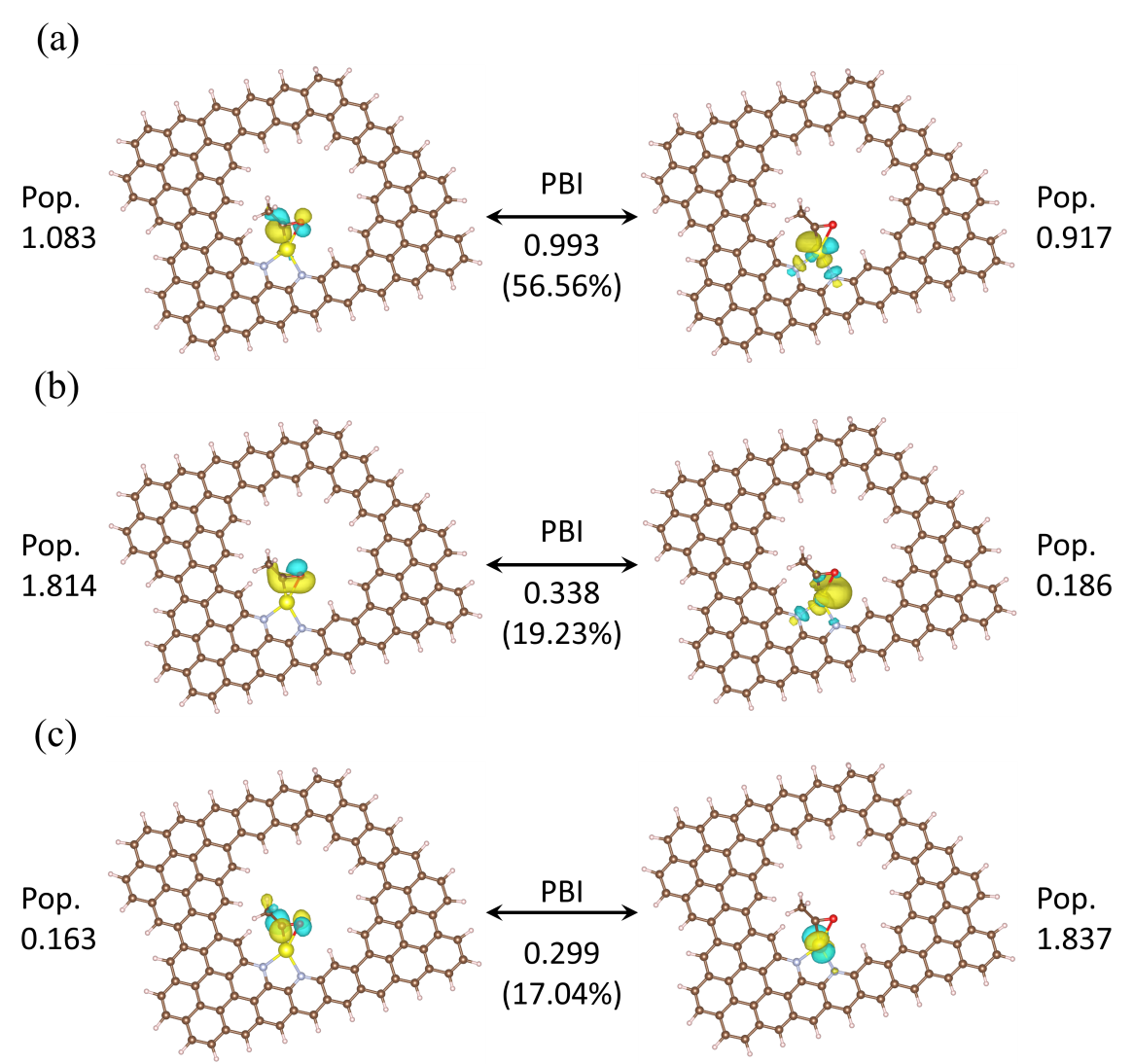


a

b

c

**Figure S21.** PIO analysis of the interactions between adsorbed CH_3_CO and substrate of Pt_1_/NCNC. Isovalue 0.05 (e/bohr^3^)^1/2^ (a) Dominant PIOs for the interaction, consisting of the $d_{{x^{2}-y}^{2}}$ orbital of Pt and the out-of-phase combinations of $\text{C}_{{sp}^{2}}$ and $\text{O}_{{sp}^{2}}$, contribute 56.56% interaction to the total interactions, suggesting the formation of covalent bonds (b) the second interactions between adsorbed CH_3_CO and substrate of Pt_1_/NCNC, contributed by the $d_{z^{2}}$ mixing with $s$ as well as $p_{z}$ orbitals of Pt and the in-phase combinations of $\text{C}_{{sp}^{2}}$ and $\text{O}_{{sp}^{2}}$; (c) the third principal interactions between adsorbed CH_3_CO and substrate of Pt_1_/NCNC, consist of the $d_{yz}$ orbital of Pt and anti-bonding orbital of C=O, corresponding to the back-donation from Pt to CH_3_CO group. The populations (occupation numbers) are given as Pop near each PIO. The PIO-based bond indices (abbreviated as PBI) and their contribution (as %) to the total interactions between two fragments (the contributions of all PIOs sum up to 100%) are given near the arrow. Here, a large PBI indicates a stronger interaction, which also has a higher contribution to the total interactions.


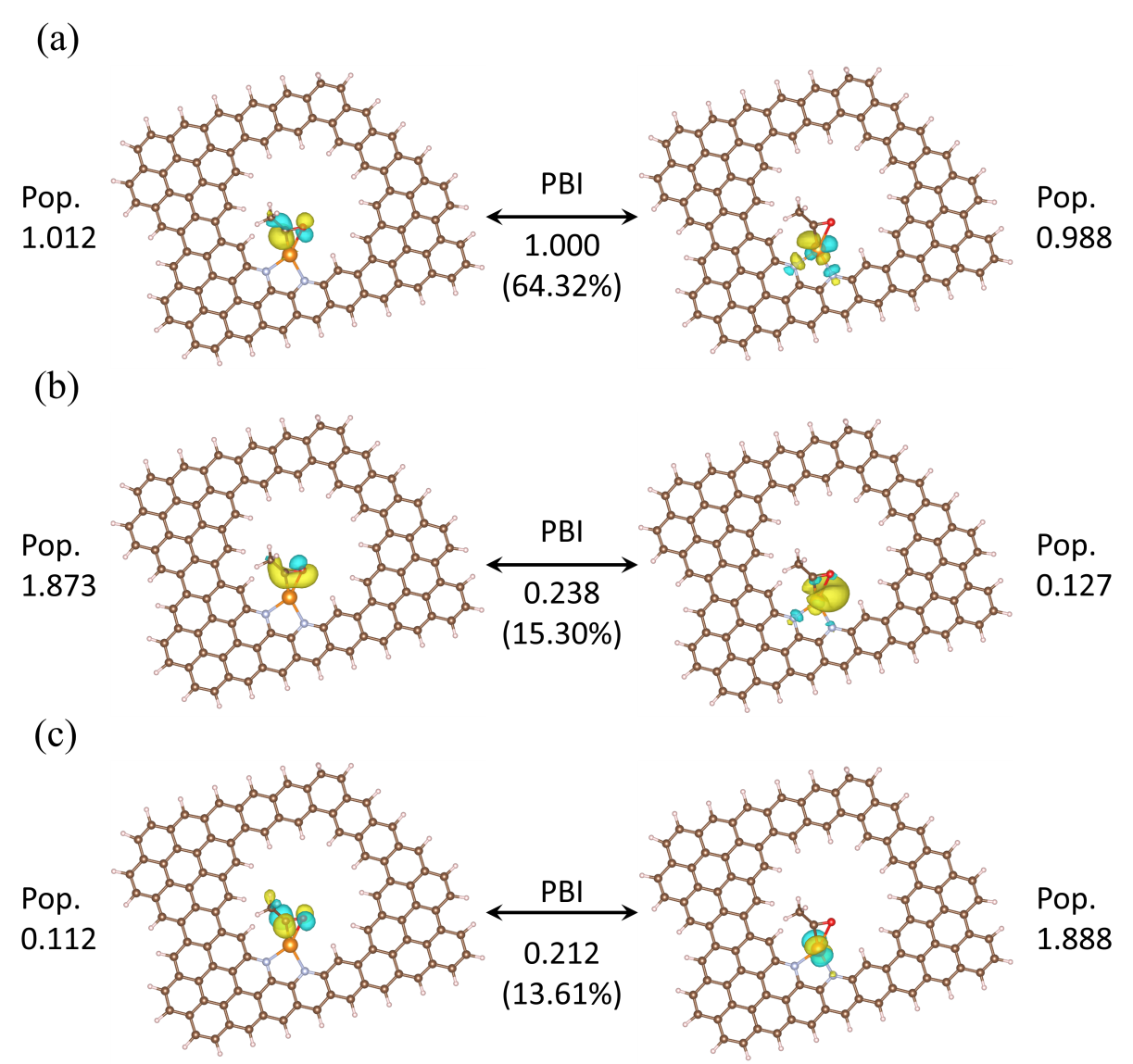


a

b

c

**Figure S22.** PIO analysis of the interactions between adsorbed CH_3_CO and substrate of Pd_1_/NCNC. Isovalue 0.05 (e/bohr^3^)^1/2^ (a) Dominant PIOs for the interaction, consisting of the $d_{{x^{2}-y}^{2}}$ orbital of Pd and the out-of-phase combinations of $\text{C}_{{sp}^{2}}$ and $\text{O}_{{sp}^{2}}$, contribute 64.32% interaction to the total interactions, suggesting the formation of covalent bonds (b) the second interactions between adsorbed CH_3_CO and substrate of Pd_1_/NCNC, contributed by the $d_{z^{2}}$ mixing with $s$ as well as $p_{z}$ orbitals of Pd and the in-phase combinations of $\text{C}_{{sp}^{2}}$ and $\text{O}_{{sp}^{2}}$; (c) the third principal interactions between adsorbed CH_3_CO and substrate of Pd_1_/NCNC, consist of the $d_{yz}$ orbital of Pd and *anti*-bonding orbital of C=O, corresponding to the back-donation from Pd to CH_3_CO group. The populations (occupation numbers) are given as Pop near each PIO. The PIO-based bond indices (abbreviated as PBI) and their contribution (as %) to the total interactions between two fragments (the contributions of all PIOs sum up to 100%) are given near the arrow. Here, a large PBI indicates a stronger interaction, which also has a higher contribution to the total interactions.


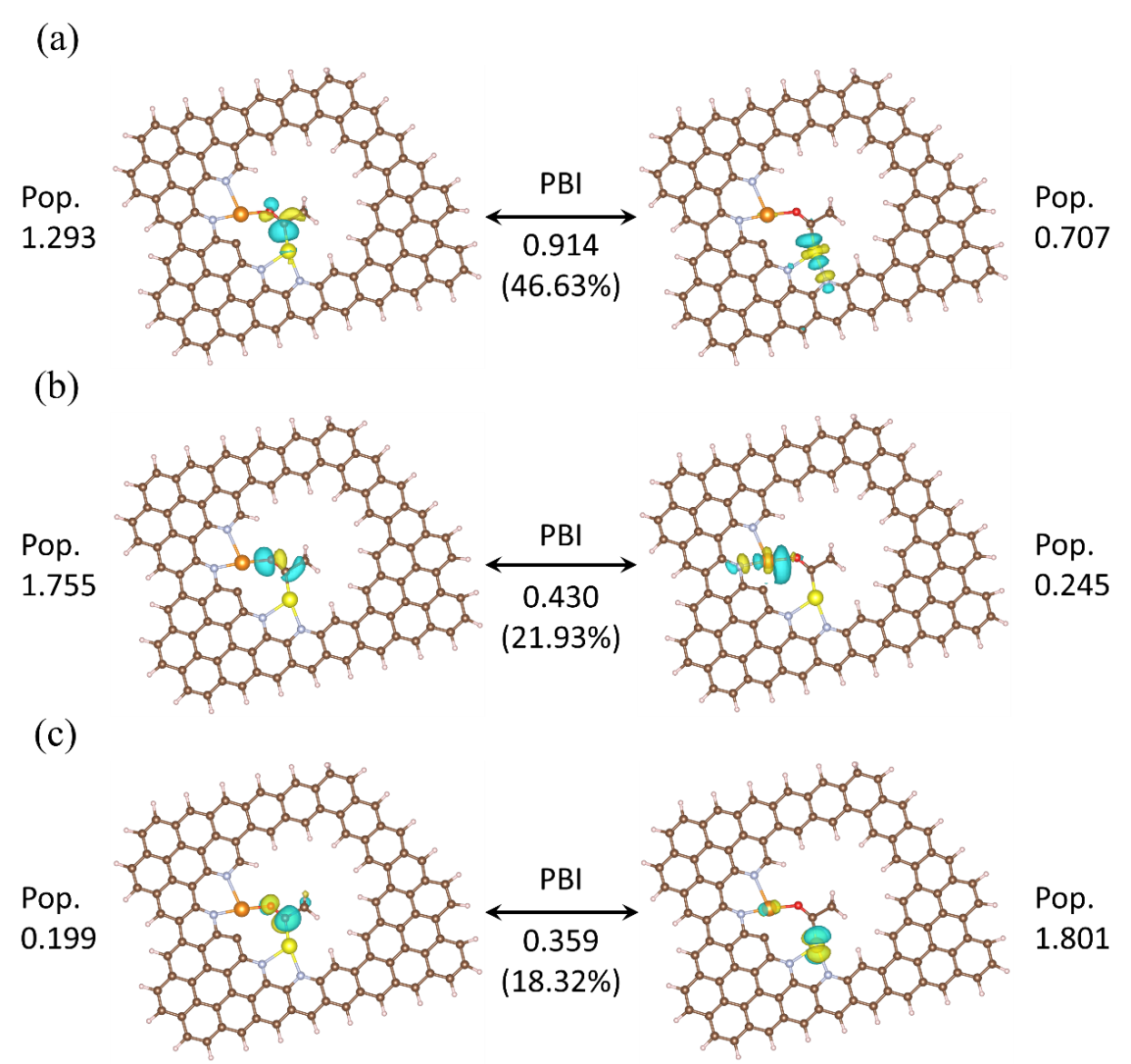


a

b

c

**Figure S23.** PIO analysis of the interactions between adsorbed CH_3_CO and substrate of Pt_1_Pd_1_/NCNC. Isovalue 0.05 (e/bohr^3^)^1/2^. (a) Dominant PIOs for the interaction consisting of the $d_{z^{2}}$ orbital of Pt and the $\text{C}_{{sp}^{2}}$ orbital of CH_3_CO contribute 46.63% interaction to the total interactions, suggesting the formation of covalent bonds (b) the second interactions between adsorbed CH_3_CO and substrate of Pt_1_Pd_1_/NCNC, contributed by the $d_{z^{2}}$ mixing with $s$ as well as $p_{z}$ orbitals of Pd and the $p$ orbital of O; (c) the third principal interactions between adsorbed CH_3_CO and substrate of Pt_1_Pd_1_/NCNC, consisting of the $d_{yz}$ orbital of Pt as well as Pd, and anti-bonding orbital of C=O, corresponding to the back-donation from Pt, Pd to CH_3_CO group. The populations (occupation numbers) are given as Pop near each PIO. The PIO-based bond indices (abbreviated as PBI) and their contribution (as %) to the total interactions between two fragments (the contributions of all PIOs sum up to 100%) are given near the arrow. Here, a large PBI indicates a stronger interaction, which also has a higher contribution to the total interactions.


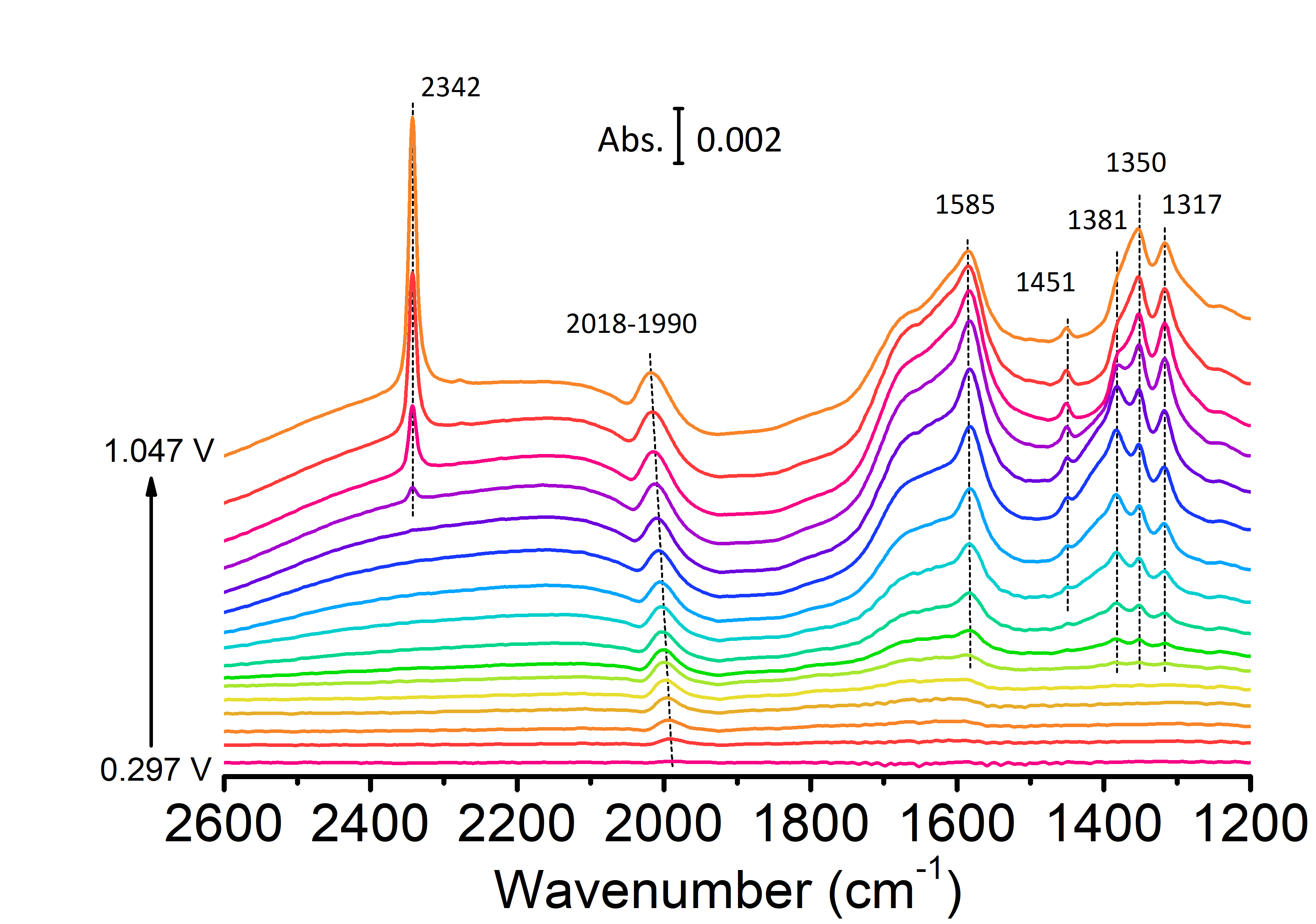


**Figure S24.** *Operando* IRRAS recorded during the MOR on 20 wt% commercial Pt/C.


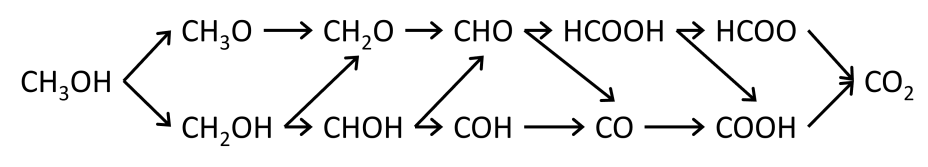


**Figure S25.** Reaction diagram for oxidation of methanol oxidation towards CO_2_.

**Table S1.** EXAFS parameters of Pt_1_Pd_1_/NCNC and Pt_1_/NCNC.

| Sample | Shell | N | R (Å) | σ^2^ (10^-3^Å^2^) |
| --- | --- | --- | --- | --- |
| Pt_1_Pd_1_/NCNC | Pt‒N | 2.0 | 2.1713±0.0206 | 1.79±0.26 |
|  | Pt‒C | 2.0 | 2.8466±0.0114 | 31.6±8.75 |
|  | Pt‒Pd | 1.1 | 4.0994±0.0084 | 15.0±3.41 |
| Pt_1_/NCNC | Pt‒N | 2.0 | 2.0497±0.0125 | 0.1±0.09 |
|  | Pt‒C | 2.0 | 2.7929±0.0076 | 18.35±4.15 |

**Table S2.** EXAFS parameters of Pt_1_Pd_1_/NCNC and Pt_1_/NCNC.

| Sample | Shell | N | R (Å) | σ^2^ (10^-3^Å^2^) |
| --- | --- | --- | --- | --- |
| Pt_1_Pd_1_/NCNC | Pd‒N_1_ | 1.0 | 2.0122±0.0103 | 0.001±0.001 |
|  | Pd‒N_2_ | 1.0 | 2.4390±0.0110 | 0.001±0.001 |
|  | Pd‒C_1_ | 1.0 | 2.1141±0.0103 | 0.001±0.001 |
|  | Pd‒C_2_ | 1.0 | 2.4761±0.0113 | 0.001±0.001 |
|  | Pd‒Pt | 1.1 | 4.1094±0.0103 | 15.40±1.36 |
| Pd_1_/NCNC | Pd‒N | 2.0 | 2.1223±0.0116 | 17.69±1.57 |
|  | Pd‒C | 2.0 | 2.8649±0.0109 | 51.06±10.68 |

**Table S3.** EOR activities of Pt-based electrocatalysts in alkaline media.

| Catalyst | Electrolyte | Sweep rate  (mV s^‒1^) | All metal normalized mass activity  (mA mg^‒1^) | Chronoamperometric  stability | Reference |
| --- | --- | --- | --- | --- | --- |
| Pt_1_Pd_1_/NCNC | **0.1M KOH + 1M ethanol** | **50** | **2692.5 ± 50.4^a^** | **73.1% activity after 3600 s; 44.7% activity after 20 h** | **This study** |
| *o*-PdH0.43@Pt | 0.1M KOH + 1M ethanol | 50 | 1680^a^ | ~2% activity after 3600 s | [^22^](#_ENREF_22) |
| PdPt nanowires | 0.5M NaOH + 1M ethanol | 50 | ~940^a^ | ~2% activity after 200 s | [^23^](#_ENREF_23) |
| Corallite-like PtPd  nanostructures | 0.5M KOH + 1M ethanol | 50 | ~1080^a^ | 67.76% activity after 2000 s | [^24^](#_ENREF_24) |
| PtPd/RGO | 1M KOH + 1M ethanol | 50 | 300^a^ | 6.7% activity after 4000 s | [^25^](#_ENREF_25) |
| Pt–Pd (1:3)/RGO/GC | 1M KOH + 1M ethanol | 50 | 1486.7 ^a^ | 14.2% activity after 3600 s | [^26^](#_ENREF_26) |
| PtPd NFs-RGO/GCE | 1M KOH + 0.5M ethanol | 50 | ~610^a^ | 1.6% activity after 4000 s | [^27^](#_ENREF_27) |
|  |  |  |  |  |  |
| Pt/α-PtO*_x_*/WO_3_ | 0.1M KOH + 0.5M ethanol | 50 | 2760^b^ | 22.88% activity after 3600 s | [^28^](#_ENREF_28) |
| PdAg nanodendrites | 1M KOH + 1M ethanol | 50 | 2630^b^ | 29.7% activity after 10000 s | [^29^](#_ENREF_29) |
| Pd nanowire  @crystalline CuO*_x_* | 1M KOH + 1M ethanol | 50 | ~500^b^ | N.A | [^30^](#_ENREF_30) |
| PtMo/C | 0.5M KOH + 1M ethanol | 50 | 1000^b^ | ~5.6% activity after 900 s | [^31^](#_ENREF_31) |
| Multilayer m-Pd/PdPt  film | 1M KOH + 0.5M ethanol | 50 | 1040^b^ | ~25% activity after 3000 s | [^32^](#_ENREF_32) |
| PdCo nanotube  arrays on carbon  cloth | 1M KOH + 1M ethanol | 50 | 1500^b^ | ~16% activity after 550 s | [^33^](#_ENREF_33) |
| Pd/Ni(OH)_2_/rGO | 1M KOH + 1M ethanol | 50 | 1550^b^ | ~23.4% activity after 20000 s | [^34^](#_ENREF_34) |
| PdAu tubes | 1M KOH + 1M ethanol | 50 | ~2100^b^ | ~16.7% activity after 2000 s | [^35^](#_ENREF_35) |
| PtCu nanowires | 1M KOH + 1M ethanol | 50 | 2100^b^ | ~5.4% activity after 4000 s | [^36^](#_ENREF_36) |

Notes: a: the mass activity is normalized relative to the combined Pd and Pt mass loading. b: the mass activity is normalized to the mass loading of either Pt or Pd.

**Table S4.** Calculated free energies (eV) for intermediates on different catalysts.

|  | CH_3_CH_2_OH | CH_3_CH_2_O | CH_3_CHO | CH_3_CHOH | CH_3_CO | CH_3_COH | CH_3_COOH |
| --- | --- | --- | --- | --- | --- | --- | --- |
| Pt_1_/NCNC | ‒1.21 | ‒1.43 | ‒2.41 | ‒1.97 | ‒2.21 | ‒1.58 | ‒0.92 |
| Pd_1_/NCNC | ‒0.89 | ‒0.94 | ‒1.64 | ‒1.14 | ‒1.35 | ‒0.41 | ‒0.76 |
| Pt_1_Pd_1_/NCNC | ‒1.63 | ‒2.21 | ‒1.14 | ‒2.15 | ‒2.69 | ‒2.16 | ‒2.78 |

**Table S5.** Computed vibrational frequencies (cm^‒1^) of CH_3_CH_2_OH adsorbed on different substrates.

| Vibration mode | Pt_1_/NCNC | Pd_1_/NCNC | Pt_1_Pd_1_/NCNC |
| --- | --- | --- | --- |
| 1 | 3017.13 | 3071.00 | 3055.66 |
| 2 | 2935.04 | 3012.07 | 3034.93 |
| 3 | 2915.86 | 2945.22 | 2988.25 |
| 4 | 2831.72 | 2917.88 | 2966.79 |
| 5 | 2593.03 | 2691.19 | 2650.20 |
| 6 | 2327.94 | 2589.00 | 2418.41 |
| 7 | 1535.84 | 1482.61 | 1444.10 |
| 8 | 1470.77 | 1465.29 | 1425.33 |
| 9 | 1468.47 | 1420.96 | 1402.31 |
| 10 | 1343.45 | 1368.61 | 1367.98 |
| 11 | 1297.85 | 1293.52 | 1351.69 |
| 12 | 1232.41 | 1252.14 | 1325.40 |
| 13 | 1203.59 | 1217.18 | 1251.08 |
| 14 | 1108.15 | 1121.26 | 1105.81 |
| 15 | 1061.59 | 1053.24 | 1058.87 |
| 16 | 961.69 | 989.89 | 1020.43 |
| 17 | 863.76 | 866.94 | 925.98 |
| 18 | 801.24 | 813.64 | 850.10 |
| 19 | 581.32 | 485.42 | 784.19 |
| 20 | 523.12 | 433.87 | 446.31 |
| 21 | 372.24 | 379.56 | 426.79 |
| 22 | 298.12 | 235.80 | 273.87 |
| 23 | 158.74 | 127.43 | 218.03 |
| 24 | 110.90 | 116.20 | 182.71 |
| 25 | 89.74 | 66.40 | 129.71 |
| 26 | 77.80 | 36.90 | 99.91 |
| 27 | 40.05 | 13.99 | 55.03 |

**Table S6.** Computed vibrational frequencies (cm^‒1^) of CH_3_CH_2_O adsorbed on different substrates.

| Vibration mode | Pt_1_/NCNC | Pd_1_/NCNC | Pt_1_Pd_1_/NCNC |
| --- | --- | --- | --- |
| 1 | 3061.37 | 3042.86 | 3053.97 |
| 2 | 3048.15 | 3024.26 | 3020.06 |
| 3 | 2968.61 | 2951.62 | 2959.67 |
| 4 | 2954.42 | 2838.10 | 2946.96 |
| 5 | 2917.83 | 2817.29 | 2888.50 |
| 6 | 1454.98 | 1447.80 | 1452.87 |
| 7 | 1432.33 | 1422.32 | 1434.93 |
| 8 | 1430.25 | 1420.88 | 1423.82 |
| 9 | 1355.52 | 1336.18 | 1349.78 |
| 10 | 1313.53 | 1305.75 | 1323.63 |
| 11 | 1241.21 | 1234.65 | 1252.45 |
| 12 | 1107.33 | 1111.84 | 1130.71 |
| 13 | 1067.36 | 1073.61 | 1063.90 |
| 14 | 1019.54 | 1026.17 | 1021.80 |
| 15 | 889.76 | 866.94 | 864.18 |
| 16 | 775.62 | 771.81 | 778.60 |
| 17 | 558.02 | 493.74 | 504.18 |
| 18 | 329.69 | 348.33 | 398.68 |
| 19 | 267.06 | 241.89 | 247.82 |
| 20 | 201.69 | 182.45 | 237.49 |
| 21 | 152.73 | 93.96 | 151.83 |
| 22 | 108.25 | 65.36 | 118.67 |
| 23 | 74.26 | 36.31 | 60.71 |
| 24 | 54.13 | 6.31 | 42.76 |

**Table S7.** Computed vibrational frequencies (cm^‒1^) of CH_3_CHO adsorbed on different substrates.

| Vibration mode | Pt_1_/NCNC | Pd_1_/NCNC | Pt_1_Pd_1_/NCNC |
| --- | --- | --- | --- |
| 1 | 3059.28 | 3058.35 | 3065.06 |
| 2 | 3014.89 | 3010.89 | 3012.16 |
| 3 | 2938.23 | 2930.03 | 2992.58 |
| 4 | 2887.13 | 2862.08 | 2928.04 |
| 5 | 1437.23 | 1435.78 | 1420.11 |
| 6 | 1427.02 | 1425.25 | 1409.11 |
| 7 | 1346.54 | 1359.12 | 1356.21 |
| 8 | 1328.23 | 1338.83 | 1331.23 |
| 9 | 1154.70 | 1230.23 | 1219.60 |
| 10 | 1076.19 | 1070.64 | 1088.27 |
| 11 | 1049.86 | 1023.57 | 976.85 |
| 12 | 922.03 | 872.49 | 883.37 |
| 13 | 866.58 | 864.13 | 683.12 |
| 14 | 564.42 | 483.42 | 498.86 |
| 15 | 475.41 | 457.93 | 390.48 |
| 16 | 436.71 | 389.24 | 310.72 |
| 17 | 233.60 | 206.76 | 283.49 |
| 18 | 188.40 | 179.59 | 156.02 |
| 19 | 137.49 | 116.74 | 136.30 |
| 20 | 94.17 | 89.91 | 104.00 |
| 21 | 66.52 | 56.10 | 48.77 |

**Table S8.** Computed vibrational frequencies (cm^‒1^) of CH_3_CHOH adsorbed on different substrates.

| Vibration mode | Pt_1_/NCNC | Pd_1_/NCNC | Pt_1_Pd_1_/NCNC |
| --- | --- | --- | --- |
| 1 | 3639.11 | 3656.05 | 3684.22 |
| 2 | 3049.93 | 3050.43 | 3039.00 |
| 3 | 3028.19 | 3019.63 | 3006.41 |
| 4 | 2986.22 | 3001.35 | 2974.69 |
| 5 | 2951.24 | 2946.64 | 2918.10 |
| 6 | 1433.97 | 1431.09 | 1437.09 |
| 7 | 1430.60 | 1427.43 | 1422.37 |
| 8 | 1355.72 | 1356.88 | 1335.17 |
| 9 | 1313.08 | 1330.20 | 1287.52 |
| 10 | 1190.69 | 1209.02 | 1217.53 |
| 11 | 1131.03 | 1112.86 | 1076.70 |
| 12 | 1031.24 | 1049.82 | 1049.48 |
| 13 | 963.09 | 982.81 | 973.31 |
| 14 | 943.21 | 903.92 | 920.76 |
| 15 | 751.01 | 810.28 | 686.49 |
| 16 | 681.67 | 607.21 | 602.63 |
| 17 | 529.74 | 506.31 | 508.92 |
| 18 | 410.06 | 415.38 | 492.32 |
| 19 | 365.79 | 338.08 | 294.06 |
| 20 | 239.67 | 222.05 | 259.09 |
| 21 | 206.12 | 209.19 | 241.43 |
| 22 | 121.53 | 112.84 | 211.67 |
| 23 | 85.69 | 85.67 | 120.55 |
| 24 | 58.50 | 33.89 | 52.86 |

**Table S9.** Computed vibrational frequencies (cm^‒1^) of CH_3_CO adsorbed on different substrates.

| Vibration mode | Pt_1_/NCNC | Pd_1_/NCNC | Pt_1_Pd_1_/NCNC |
| --- | --- | --- | --- |
| 1 | 3095.17 | 3100.65 | 3046.46 |
| 2 | 3042.32 | 3040.32 | 3022.03 |
| 3 | 2969.96 | 2971.96 | 2937.02 |
| 4 | 1522.01 | 1598.33 | 1439.29 |
| 5 | 1424.42 | 1421.47 | 1410.82 |
| 6 | 1409.69 | 1403.88 | 1390.35 |
| 7 | 1322.52 | 1314.92 | 1300.45 |
| 8 | 1126.51 | 1087.56 | 1047.91 |
| 9 | 968.90 | 969.63 | 943.74 |
| 10 | 942.63 | 928.02 | 902.52 |
| 11 | 570.50 | 549.63 | 654.68 |
| 12 | 447.61 | 427.47 | 483.48 |
| 13 | 365.42 | 330.36 | 396.24 |
| 14 | 289.66 | 266.40 | 303.10 |
| 15 | 144.34 | 136.13 | 214.71 |
| 16 | 123.61 | 112.62 | 167.58 |
| 17 | 115.16 | 92.13 | 89.65 |
| 18 | 65.99 | 52.46 | 59.13 |

**Table S10.** Computed vibrational frequencies (cm^‒1^) of CH_3_COH adsorbed on different substrates.

| Vibration mode | Pt_1_/NCNC | Pd_1_/NCNC | Pt_1_Pd_1_/NCNC |
| --- | --- | --- | --- |
| 1 | 3579.65 | 3487.97 | 3649.76 |
| 2 | 3067.66 | 3072.92 | 3082.46 |
| 3 | 2968.58 | 2972.39 | 2983.15 |
| 4 | 2903.66 | 2901.10 | 2908.61 |
| 5 | 1428.72 | 1426.31 | 1412.62 |
| 6 | 1416.73 | 1410.79 | 1409.11 |
| 7 | 1330.86 | 1320.09 | 1324.00 |
| 8 | 1313.46 | 1305.74 | 1254.87 |
| 9 | 1160.13 | 1178.41 | 1044.73 |
| 10 | 1029.63 | 1015.19 | 1019.69 |
| 11 | 952.20 | 944.18 | 953.63 |
| 12 | 942.73 | 922.43 | 801.02 |
| 13 | 574.20 | 550.63 | 707.77 |
| 14 | 483.44 | 531.84 | 562.40 |
| 15 | 445.81 | 426.53 | 497.81 |
| 16 | 319.68 | 282.85 | 323.85 |
| 17 | 262.20 | 224.43 | 297.46 |
| 18 | 169.29 | 116.34 | 263.98 |
| 19 | 83.18 | 56.28 | 151.51 |
| 20 | 64.20 | 36.28 | 120.89 |
| 21 | 28.82 | 11.71 | 84.25 |

**Table S11.** Computed vibrational frequencies (cm^‒1^) of CH_3_COOH adsorbed on different substrates.

| Vibration mode | Pt_1_/NCNC | Pd_1_/NCNC | Pt_1_Pd_1_/NCNC |
| --- | --- | --- | --- |
| 1 | 3664.56 | 3679.34 | 3655.26 |
| 2 | 3100.62 | 3061.06 | 3085.91 |
| 3 | 3033.68 | 3035.46 | 3046.12 |
| 4 | 2968.11 | 2956.05 | 2964.44 |
| 5 | 1649.73 | 1537.36 | 1429.63 |
| 6 | 1430.41 | 1432.38 | 1425.01 |
| 7 | 1413.40 | 1415.31 | 1353.86 |
| 8 | 1353.31 | 1333.58 | 1315.08 |
| 9 | 1285.13 | 1076.88 | 1120.97 |
| 10 | 1167.59 | 1036.43 | 1045.86 |
| 11 | 1010.19 | 985.34 | 1032.70 |
| 12 | 984.68 | 884.34 | 901.46 |
| 13 | 863.71 | 583.03 | 684.62 |
| 14 | 627.90 | 573.06 | 639.03 |
| 15 | 561.38 | 548.20 | 534.26 |
| 16 | 476.18 | 442.02 | 483.74 |
| 17 | 456.83 | 389.10 | 439.46 |
| 18 | 234.67 | 244.83 | 418.34 |
| 19 | 178.55 | 202.81 | 279.11 |
| 20 | 130.50 | 185.60 | 244.95 |
| 21 | 102.80 | 182.78 | 205.93 |
| 22 | 61.78 | 87.83 | 182.17 |
| 23 | 32.50 | 79.84 | 121.10 |
| 24 | 15.72 | 61.84 | 80.31 |

**Table S12.** Calculated d-band centers (eV) relative to the Fermi-level.

|  | Pt_1_/NCNC | Pd_1_/NCNC | Pt_1_Pd_1_/NCNC |
| --- | --- | --- | --- |
| Pt | -2.255 |  | -2.027 |
| Pd |  | -1.792 | -1.841 |

**Table S13.** Computed vibrational frequencies (cm^‒1^) of CH_3_OH, CH_2_OH, HCOOH, CH_3_O, CH_2_O, and CHOH intermediates on Pt_1_Pd_1_/NCNC.

| Mode | CH_3_OH | CH_2_OH | HCOOH | CH_3_O | CH_2_O | CHOH |
| --- | --- | --- | --- | --- | --- | --- |
| 1 | 3063.93 | 3690.67 | 3653.08 | 2988.40 | 3098.15 | 3666.56 |
| 2 | 2998.27 | 3031.03 | 2978.88 | 2955.61 | 2973.85 | 2972.34 |
| 3 | 2920.57 | 2887.42 | 1342.61 | 2891.00 | 1436.92 | 1355.11 |
| 4 | 2311.46 | 1403.96 | 1214.08 | 1434.93 | 1181.20 | 1113.61 |
| 5 | 1441.57 | 1260.39 | 1124.46 | 1425.16 | 1130.04 | 899.44 |
| 6 | 1427.85 | 1185.16 | 1035.56 | 1403.36 | 788.64 | 841.16 |
| 7 | 1411.37 | 1090.58 | 747.22 | 1140.99 | 547.90 | 698.61 |
| 8 | 1385.23 | 806.81 | 650.36 | 1114.89 | 404.66 | 625.31 |
| 9 | 1130.67 | 684.17 | 555.73 | 1018.43 | 288.09 | 378.18 |
| 10 | 1112.30 | 596.16 | 485.00 | 476.47 | 256.33 | 314.34 |
| 11 | 998.44 | 478.02 | 430.13 | 276.98 | 201.47 | 259.67 |
| 12 | 931.73 | 376.83 | 299.03 | 170.64 | 77.11 | 128.72 |
| 13 | 434.44 | 280.69 | 230.41 | 145.80 |  |  |
| 14 | 228.03 | 164.32 | 172.32 | 90.94 |  |  |
| 15 | 189.13 | 114.57 | 108.52 | 44.81 |  |  |
| 16 | 119.55 |  |  |  |  |  |
| 17 | 42.88 |  |  |  |  |  |
| 18 | 38.04 |  |  |  |  |  |

**Table S14.** Computed vibrational frequencies (cm^‒1^) of COOH, HCOO, CHO, CO_2_, COH, and CO intermediates on Pt_1_Pd_1_/NCNC.

| Mode | COOH | HCOO | CHO | CO_2_ | COH | CO |
| --- | --- | --- | --- | --- | --- | --- |
| 1 | 3563.39 | 2878.69 | 2700.08 | 1586.12 | 3594.39 | 1709.94 |
| 2 | 1525.85 | 1274.44 | 1443.18 | 1187.63 | 1284.89 | 615.64 |
| 3 | 1272.46 | 1221.38 | 1282.10 | 766.27 | 1145.70 | 484.51 |
| 4 | 1076.59 | 1104.39 | 760.96 | 564.20 | 611.45 | 426.15 |
| 5 | 658.46 | 773.20 | 573.27 | 434.23 | 461.00 | 294.44 |
| 6 | 566.90 | 654.31 | 420.87 | 385.41 | 384.82 | 121.84 |
| 7 | 420.53 | 444.81 | 349.22 | 271.30 | 310.03 |  |
| 8 | 382.22 | 330.93 | 268.19 | 190.88 | 269.85 |  |
| 9 | 299.96 | 278.58 | 96.19 | 113.14 | 103.60 |  |
| 10 | 200.92 | 202.07 |  |  |  |  |
| 11 | 129.41 | 176.83 |  |  |  |  |
| 12 | 93.36 | 101.63 |  |  |  |  |

**References:**

(1) Chen, S.; Bi, J.; Zhao, Y.; Yang, L.; Zhang, C.; Ma, Y.; Wu, Q.; Wang, X.; Hu, Z. Nitrogen-doped carbon nanocages as efficient metal-free electrocatalysts for oxygen reduction reaction. *Adv. Mater.* **2012**, *24* (41), 5593-5597

(2) Ravel, B.; Newville, M. ATHENA, ARTEMIS, HEPHAESTUS: data analysis for X-ray absorption spectroscopy using IFEFFIT. *J. Synchrotron Rad.* **2005**, *12* (4), 537-541

(3) Anantharaj, S.; Karthik, P. E.; Noda, S. The significance of properly reporting turnover frequency in electrocatalysis research. *Angew. Chem. Int. Ed.* **2021**, *60* (43), 23051-23067

(4) Malko, D.; Kucernak, A.; Lopes, T. In situ electrochemical quantification of active sites in Fe–N/C non-precious metal catalysts. *Nat. Commun.* **2016**, *7* (1), 13285

(5) Gong, M.; Mehmood, A.; Ali, B.; Nam, K.-W.; Kucernak, A. Oxygen reduction reaction activity in non-precious single-atom (M–N/C) catalysts─contribution of metal and carbon/nitrogen framework-based sites. *ACS Catal.* **2023**, *13* (10), 6661-6674

(6) Kresse, G.; Furthmüller, J. Efficient iterative schemes for ab initio total-energy calculations using a plane-wave basis set. *Phys. Rev. B* **1996**, *54* (16), 11169-11186

(7) Kresse, G.; Furthmüller, J. Efficiency of ab-initio total energy calculations for metals and semiconductors using a plane-wave basis set. *Comput. Mater. Sci.* **1996**, *6* (1), 15-50

(8) Blöchl, P. E. Projector augmented-wave method. *Phys. Rev. B* **1994**, *50* (24), 17953-17979

(9) Perdew, J. P.; Burke, K.; Ernzerhof, M. Generalized gradient approximation made simple. *Phys. Rev. Lett.* **1996**, *77* (18), 3865-3868

(10) Zhang, Z.; Chen, Y.; Zhou, L.; Chen, C.; Han, Z.; Zhang, B.; Wu, Q.; Yang, L.; Du, L.; Bu, Y.; et al. The simplest construction of single-site catalysts by the synergism of micropore trapping and nitrogen anchoring. *Nat. Commun.* **2019**, *10* (1), 1657

(11) Grimme, S.; Antony, J.; Ehrlich, S.; Krieg, H. A consistent and accurate ab initio parametrization of density functional dispersion correction (DFT-D) for the 94 elements H-Pu. *J. Chem. Phys.* **2010**, *132* (15), 154104

(12) Ferrin, P.; Mavrikakis, M. Structure sensitivity of methanol electrooxidation on transition metals. *J. Am. Chem. Soc.* **2009**, *131* (40), 14381-14389

(13) Wang, V.; Xu, N.; Liu, J.-C.; Tang, G.; Geng, W.-T. VASPKIT: A user-friendly interface facilitating high-throughput computing and analysis using VASP code. *Comput. Phys. Commun.* **2021**, *267*, 108033

(14) Nørskov, J. K.; Rossmeisl, J.; Logadottir, A.; Lindqvist, L.; Kitchin, J. R.; Bligaard, T.; Jónsson, H. Origin of the overpotential for oxygen reduction at a fuel-cell cathode. *J. Phys. Chem. B* **2004**, *108* (46), 17886-17892

(15) Frisch, M.; Trucks, G.; Schlegel, H.; Scuseria, G.; Robb, M.; Cheeseman, J.; Scalmani, G.; Barone, V.; Mennucci, B.; Petersson, G.; et al. Gaussian 09 (Revision A02). *Gaussian Inc. Wallingford CT* **2009**,

(16) Hay, P. J.; Wadt, W. R. Ab *initio* effective core potentials for molecular calculations. Potentials for the transition metal atoms Sc to Hg. *J. Chem. Phys.* **1985**, *82* (1), 270-283

(17) Igel-Mann, G.; Stoll, H.; Preuss, H. Pseudopotentials for main group elements (IIIa through VIIa). *Mol. Phys.* **1988**, *65* (6), 1321-1328

(18) Sun, H.; Li, L.; Chen, Y.; Kim, H.; Xu, X.; Guan, D.; Hu, Z.; Zhang, L.; Shao, Z.; Jung, W. Boosting ethanol oxidation by NiOOH-CuO nano-heterostructure for energy-saving hydrogen production and biomass upgrading. *Appl. Catal. B* **2023**, *325*, 122388

(19) Chen, Y.; Xia, M.; Zhou, C.; Zhang, Y.; Zhou, C.; Xu, F.; Feng, B.; Wang, X.; Yang, L.; Hu, Z.; et al. Hierarchical dual Single-atom catalysts with coupled CoN_4_ and NiN_4_ moieties for industrial-level CO_2_ electroreduction to syngas. *ACS Nano* **2023**, *17* (21), 22095-22105

(20) Chang, J.; Wang, G.; Wang, M.; Wang, Q.; Li, B.; Zhou, H.; Zhu, Y.; Zhang, W.; Omer, M.; Orlovskaya, N.; et al. Improving Pd–N–C fuel cell electrocatalysts through fluorination-driven rearrangements of local coordination environment. *Nat. Energy* **2021**, *6* (12), 1144-1153

(21) Chang, J.; Wang, G.; Chang, X.; Yang, Z.; Wang, H.; Li, B.; Zhang, W.; Kovarik, L.; Du, Y.; Orlovskaya, N.; et al. Interface synergism and engineering of Pd/Co@N-C for direct ethanol fuel cells. *Nat. Commun.* **2023**, *14* (1), 1346

(22) Liu, G.; Zhou, W.; Ji, Y.; Chen, B.; Fu, G.; Yun, Q.; Chen, S.; Lin, Y.; Yin, P.-F.; Cui, X.; et al. Hydrogen-intercalation-induced lattice expansion of Pd@Pt core–shell nanoparticles for highly efficient electrocatalytic alcohol oxidation. *J. Am. Chem. Soc.* **2021**, *143* (29), 11262-11270

(23) Zhu, C.; Guo, S.; Dong, S. PdM (M = Pt, Au) bimetallic alloy nanowires with enhanced electrocatalytic activity for electro-oxidation of small molecules. *Adv. Mater.* **2012**, *24* (17), 2326-2331

(24) Liu, X.-Y.; Zhang, Y.; Gong, M.-X.; Tang, Y.-W.; Lu, T.-H.; Chen, Y.; Lee, J.-M. Facile synthesis of corallite-like Pt–Pd alloy nanostructures and their enhanced catalytic activity and stability for ethanol oxidation. *J. Mater. Chem. A* **2014**, *2* (34), 13840-13844

(25) Li, S.-S.; Zheng, J.-N.; Ma, X.; Hu, Y.-Y.; Wang, A.-J.; Chen, J.-R.; Feng, J.-J. Facile synthesis of hierarchical dendritic PtPd nanogarlands supported on reduced graphene oxide with enhanced electrocatalytic properties. *Nanoscale* **2014**, *6* (11), 5708-5713

(26) Ren, F.; Wang, H.; Zhai, C.; Zhu, M.; Yue, R.; Du, Y.; Yang, P.; Xu, J.; Lu, W. Clean method for the synthesis of reduced graphene oxide-supported PtPd alloys with high electrocatalytic activity for ethanol oxidation in alkaline medium. *ACS Appl. Mater. Interfaces* **2014**, *6* (5), 3607-3614

(27) Lv, J.-J.; Wisitruangsakul, N.; Feng, J.-J.; Luo, J.; Fang, K.-M.; Wang, A.-J. Biomolecule-assisted synthesis of porous PtPd alloyed nanoflowers supported on reduced graphene oxide with highly electrocatalytic performance for ethanol oxidation and oxygen reduction. *Electrochim. Acta* **2015**, *160*, 100-107

(28) Xiao, L.; Li, G.; Yang, Z.; Chen, K.; Zhou, R.; Liao, H.; Xu, Q.; Xu, J. Engineering of amorphous PtO*_x_* interface on Pt/WO_3_ nanosheets for ethanol oxidation electrocatalysis. *Adv. Funct. Mater.* **2021**, *31* (28), 2100982

(29) Huang, W.; Kang, X.; Xu, C.; Zhou, J.; Deng, J.; Li, Y.; Cheng, S. 2D PdAg alloy nanodendrites for enhanced ethanol electroxidation. *Adv. Mater.* **2018**, *30* (11), 1706962

(30) Chen, Z.; Liu, Y.; Liu, C.; Zhang, J.; Chen, Y.; Hu, W.; Deng, Y. Engineering the metal/oxide interface of Pd nanowire@CuO*_x_* electrocatalysts for efficient alcohol oxidation reaction. *Small* **2020**, *16* (4), 1904964

(31) Pech-Rodríguez, W. J.; González-Quijano, D.; Vargas-Gutiérrez, G.; Morais, C.; Napporn, T. W.; Rodríguez-Varela, F. J. Electrochemical and *in situ* FTIR study of the ethanol oxidation reaction on PtMo/C nanomaterials in alkaline media. *Appl. Catal. B* **2017**, *203*, 654-662

(32) Jiang, B.; Li, C.; Qian, H.; Hossain, M. S. A.; Malgras, V.; Yamauchi, Y. Layer-by-layer motif architectures: programmed electrochemical syntheses of multilayer mesoporous metallic films with uniformly sized pores. *Angew. Chem. Int. Ed.* **2017**, *56* (27), 7836-7841

(33) Wang, A.-L.; He, X.-J.; Lu, X.-F.; Xu, H.; Tong, Y.-X.; Li, G.-R. Palladium–cobalt nanotube arrays supported on carbon fiber cloth as high-performance flexible electrocatalysts for ethanol oxidation. *Angew. Chem. Int. Ed.* **2015**, *54* (12), 3669-3673

(34) Huang, W.; Ma, X.-Y.; Wang, H.; Feng, R.; Zhou, J.; Duchesne, P. N.; Zhang, P.; Chen, F.; Han, N.; Zhao, F.; et al. Promoting effect of Ni(OH)_2_ on palladium nanocrystals leads to greatly improved operation durability for electrocatalytic ethanol oxidation in alkaline solution. *Adv. Mater.* **2017**, *29* (37), 1703057

(35) Cui, C.-H.; Yu, J.-W.; Li, H.-H.; Gao, M.-R.; Liang, H.-W.; Yu, S.-H. Remarkable enhancement of electrocatalytic activity by tuning the interface of Pd–Au bimetallic nanoparticle tubes. *ACS Nano* **2011**, *5* (5), 4211-4218

(36) Hong, W.; Wang, J.; Wang, E. Facile synthesis of PtCu nanowires with enhanced electrocatalytic activity. *Nano Res.* **2015**, *8* (7), 2308-2316
